# Robust Development of Gold Nanorod-Quantum Dot Assemblies for Dynamic Dual-Modal Single Nanoparticle Imaging and Tracking Applications

**DOI:** 10.1021/acsanm.5c03231

**Published:** 2025-12-08

**Authors:** Hannah L. Taysum, Aryanne L. Finnie, Aimee McKay, Alastair W. Wark

**Affiliations:** Technology and Innovation Centre, Department of Pure & Applied Chemistry, 3527 University of Strathclyde, 99 George St, Glasgow G1 1RD, U.K.

**Keywords:** gold nanorod-quantum dot conjugation, dual-modal Rayleigh scattering and fluorescence single nanoparticle imaging, nanoassembly characterization, functional polymer wrapping, dynamic multimodal imaging and tracking of suspended nanoparticles

## Abstract

The development of optical microscopy methods for the dynamic imaging and tracking of individual nanoparticles and their assemblies continues to significantly contribute to transforming colloidal particle characterization and the integration of functionalized nanoparticles into a wide range of advanced applications. The real-time imaging of freely diffusing nanoparticles in suspension typically involves a single optical modality (e.g., Rayleigh or fluorescence scattering) which limits the potential of this approach to characterize more complex nanoparticle systems that feature at least two properties which require very different detection sensitivities. However, correlated imaging methods tend to be performed for nanoparticles that are confined close to a surface. To address this, we have developed a robust approach for the preparation of functionalized gold nanorod core – quantum dot (AuNR-QD) conjugates alongside a dual-modal setup that enables correlated plasmonic-enhanced Rayleigh scattering and fluorescence imaging of these nanoassemblies in suspension. Two separate routes are reported for the preparation of the AuNR-QD conjugates, which are also polymer wrapped to enable a stable and flexible platform for further functionalization and application integration. The utilization of real-time dual-modal imaging for assessing QD conjugation at various particle concentration ratios is demonstrated and the results of both dynamic colloidal and static ex situ measurements compared.

## Graphical abstract

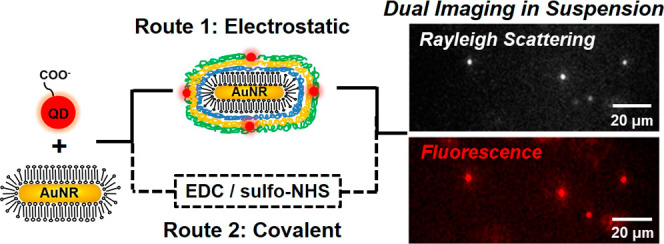

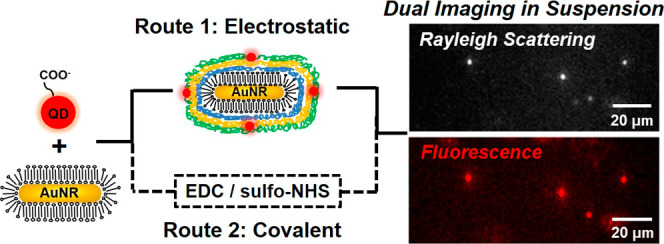

## 1 Introduction

The real-time optical detection and tracking of individual nanoparticles and nanoassemblies offers exciting measurement opportunities for a range of applications such as assay development, drug delivery and catalysis.^1–3^ More specifically, dynamic nanoparticle measurements of suspended particles typically focus only on one optical modality at a time such as Rayleigh scattering of various nanoparticle types,^4–6^ fluorescence of quantum dots (QD’s),^7–11^ exosomes,^12^ and also surface-enhanced Raman scattering of metal-nanoparticle-dyes.^13,14^ In addition, while multimodal and correlative imaging has been commonly reported for nanoparticles immobilized on a surface and in confined geometries,^15–18^ the development and application of dual-modal real-time imaging measurements of freely diffusing nanoparticles has not been explored to any significant extent.^19,20^ To address this, we describe here a robust approach for the preparation of single gold nanorod (AuNR) - QD conjugates in colloidal suspension that can be readily functionalized for further application integration. This is combined with the introduction of a novel dynamic dual-modal single particle imaging approach to enable further in-depth characterization of the nanoassemblies.

There has been significant interest in plasmon-enhanced resonance energy transfer between various metallic nanostructures and luminescent molecular dyes and quantum dots.^21–24^ Observation of metal-enhanced photoluminescence of QD’s has been investigated for both colloidal metal nanospheres^25–29^ and gold nanorods^30–33^ with controlled optical coupling strongly dependent on both the separation distance between particles and the relative wavelength ranges required for the excitation of metallic surface plasmons (SP’s) and QD excitons. Surface functionalization routes for creating suspended metal NP-QD coassemblies include the use of alkanethiol ligands,^31,32^ PEG chains,^34^ silica shell spacers^35,36^ and encapsulation,^31^ polyeletrolytes^30^ and DNA hybridization.^37^ For fundamental studies, demonstrating the robust formation of discrete single metal NP-QD assemblies that are stable in suspension has been less of a priority. Also, most applications of NP-QD systems reported have typically focused on measuring a change in plasmon-enhanced fluorescence upon binding to a specific target.^38–40^ To date, the development of discrete plasmonic NP-QD assemblies for multimodal imaging and tracking applications has been limited due to challenges in the fluorescence quenching effect. Incorporating controlled separation distances between components, achieved through the integration of polymer or organic spacers,^41,42^ can enable plasmonic-enhanced fluorescence, offering significant improvements in imaging contrast, multiplexing, and tracking capabilities for bioimaging applications.^43–45^

This work focuses on developing optically bright AuNR-QD assemblies designed to serve as Rayleigh scattering–fluorescence nanotags that are suitable for dual-modal imaging and tracking in suspension. Two different routes are highlighted for QD conjugation onto the AuNR (electrostatic adsorption and covalent coupling), with polyelectrolyte coatings used to provide control over the NR-QD spacing. Both methods were optimized for robustness and yield and also included the controlled formation of an encapsulating poly­(tannic acid) (pTA) growth layer. This addresses the significant challenge of preparing nanoassemblies such as these for subsequent application development, with pTA providing a flexible platform for a range of bioconjugation, metal complexation, and molecular encapsulation strategies.^46^ Also, while nanoparticle tracking analysis (NTA) is well-established commercially (e.g., Malvern Panalytical, Horiba) as a characterization tool for a variety of nanoparticle types, such measurements are typically limited to a single imaging modality or switching sequentially between Rayleigh scattering and fluorescence. Building on these limitations, earlier work explored the formation of SERS active nanoparticle clusters for dynamic imaging in suspension^6^ with the Rayleigh and SERS scattering videos captured sequentially. Subsequently, correlative Rayleigh and Raman/fluorescence single particle imaging in suspension has been demonstrated on a single camera split into two separately filtered channels.^19^ Here, in tandem with the development of functionalized AuNR-QD conjugates, we report a dual-modal imaging approach that enables further characterization of freely suspended individual nanoassemblies to assess the QD conjugation efficiency and compare with static measurements (optical and electron microscopy), where samples are immobilized on a surface.

## 2 Experimental Section

### 2.1 Materials

The following chemicals were sourced from Sigma-Aldrich: Gold­(III) chloride trihydrate (99%, HAuCl_4_), cetyltrimethylammonium bromide (CTAB), sodium borohydride (NaBH_4_), silver nitrate (99%, AgNO_3_), hydroquinone (99%, HQ), poly­(sodium-4-styrenesulfonate) (PSS) ∼10,000 MW, branched poly­(ethylenimine) (PEI) ∼25,000 MW, tannic acid (TA), sodium chloride (NaCl), Trizma base (tris), hydrochloric acid (HCl), sodium hydroxide (NaOH), (4-(2-hydroxyethyl)-1-piperazineethanesulfonic acid) (HEPES), *N*-(3-(dimethylamino)­propyl)-*N*′-ethylcarbodiimide hydrochloride (EDC), *N*-hydroxysulfosuccinimide sodium salt (sulfo-NHS), and ethanol (EtOH). Poly­(diallyl dimethylammonium chloride) (28 wt % in H_2_O, PDDA) ∼8500 MW was sourced from Polysciences Europe. Aqueous carboxylate core–shell QDs were sourced from ThermoFisher; Qdot655 ITKTM (8 μM, QD655). High precision microscope glass coverslips were sourced from Marienfeld Superior (size: 22 × 22 mm^2^, thickness no. 1.5) and borosilicate glass coverslips were sourced from VWR (size: 22 × 32 mm^2^, thickness no. 1). Glassware and PTFE-coated magnetic stirrer bars were soaked in aqua regia for at least 2 h, rinsed several times with deionized water, and then dried prior to synthetic work. All solutions were prepared using Millipore Milli-Q purified water.

### 2.2 Gold Nanorod Synthesis

A hydroquinone seed-mediated growth approach for AuNR synthesis described by Picciolini et al. was performed with minor adaptations.^47^

#### 2.2.1 Seed Preparation

A CTAB solution (5 mL, 0.2 M) was dissolved in water under ultrasonication at 27 °C. Separately, HAuCl_4_ solution (5 mL, 0.5 mM) dissolved in water was added to the CTAB solution under vigorous magnetic stirring. 600 μL of freshly prepared NaBH_4_ (10 mM) solution prepared in ice-cold NaOH (0.01 M) was added to the gold salt and CTAB mixture under vigorous stirring. The suspension was stirred for a further 20 min at 27 °C leading to a light yellow to light brown color change.

#### 2.2.2 Synthesis of Nanorods

A growth solution was prepared by mixing CTAB (100 mL, 0.1 M) dissolved in water with 440 mg hydroquinone at 27 °C. Separately, HAuCl_4_ (100 mL, 1 mM) prepared in water was mixed to the solution of CTAB and hydroquinone under magnetic stirring forming a colorless solution. Four mL of AgNO_3_ (4 mM) prepared in water was immediately added followed by 230 μL of the seed solution under vigorous magnetic stirring. The growth solution was stirred for a further 30 min and incubated overnight leading to a color change from colorless to maroon. The AuNRs were cleaned by two centrifugation cycles at 7400 rpm and resuspended first in water, then in 1 mM CTAB and then stored at room temperature. Prior to further use, UV–vis characterization was performed and the optical density (OD) adjusted to ∼1.8 based on the LSPR λ_max_ peak intensity. An extinction coefficient of 4.5(±0.3) × 10^9^ M^–1^ cm^–1^ was used for the NR concentrations reported here with all NR stock solutions having a λ_max_ in the region of 830 nm.^48^

### 2.3 Gold Nanorod Functionalization

Custom functionalized AuNRs were prepared by using a polyelectrolyte layer-by-layer (LbL) approach followed by a polytannic acid (pTA) growth step. After initial surface modifications with poly­(sodium-4-styrenesulfonate) (PSS), two separate routes were applied: coating the AuNRs with either poly­(diallydimethylammonium chloride) (PDDA) or poly­(ethylenimine) (PEI). For both routes, pTA growth on the passivated nanorods was achieved for further bioconjugation strategies.

#### 2.3.1 PSS-Coated Nanorods

AuNRs suspended in CTAB (100 mL, 1 mM) were vigorously stirred for 10 min to ensure particle dispersion. Twenty-five mL of PSS (10 mg mL^–1^ in 5 mM NaCl) was added dropwise, and the solution was stirred for another 10 min. The mixture underwent two centrifugation cycles at 7200 rpm with resuspensions in water.

#### 2.3.2 PDDA and PEI-Coated Nanorods

AuNRs coated with PSS were functionalized with either PDDA or PEI. A 10 mg mL^–1^ sample of the respective polyelectrolyte in 5 mM NaCl was prepared, and 5 mL was added dropwise to 20 mL of PSS-coated nanorods. The solution was stirred for 10 min followed by two centrifugation cycles at 5800 rpm with resuspensions in water, pH adjusted between 7.5 and 8.0 using 0.5 M NaOH.

#### 2.3.3 pTA Growth on Polyelectrolyte-Coated Nanorods

A tannic acid (TA) stock solution (10 mL, 0.9 mM) was prepared in Trizma base buffer (10 mM, adjusted pH 8.0, using 0.5 M HCl). Shortly after, 4 mL of TA buffered solution was aliquoted to a 10 mL of PDDA or PEI-coated nanorod solution of an OD ∼1. The mixture was shaken for 1.5–2 h at 350 rpm before two centrifugation cycles at 5500 rpm with resuspensions in HEPES buffer (10 mM, adjusted pH 8.0 using 1 M NaOH). Adjustments to the pH of polyelectrolyte-coated NRs between pH 7.5 and 8.0 were necessary to ensure slow and controlled TA polymerization. The multiple phenolic groups on TA can ionize depending on the pH. Mild alkaline solutions (pH 7.5–8) allow optimal deprotonation of the phenolic groups, increasing the negative charge of TA, allowing electrostatic interactions to positively charged surface-modified AuNRs. At this pH region, the nanoparticles will remain stable in suspension, with controlled surface coatings and improved functionality for further bioconjugation strategies.^49^

### 2.4 Preparation of NR-QD Conjugates

The NR-QD conjugates were prepared using two different methods: the electrostatic route involved a PDDA layer to facilitate interaction with carboxyl QDs via electrostatic forces, while the covalent route utilized branched PEI and a cross-linking agent for the covalent attachment of carboxyl QD655. A 1 μM QD655 stock solution was used to prepare concentration ratios ranging from [1]:[1] to [1]:[12], while the NR stock concentration remained fixed at 0.3 nM. This resulted in QD bulk concentrations of 0.5, 1, 2.5, and 5 nM.

#### 2.4.1 Electrostatic Route

The procedure described in Sections 2.2 and 2.3 was utilized to directly aliquot QD655 to PDDA-coated NRs at increasing particle concentration ratios, with respect to the NR concentration fixed at 0.3 nM, and gently shaken for 1 h in darkness. The encapsulation of QDs within the nanostructures was achieved by subsequent pTA growth, as outlined in Section 2.3. The centrifugation cycles were performed at reduced speeds (4500 rpm for 20 min) to prevent QD clustering and particle aggregation in colloidal solution. A minimum of three wash cycles was required to remove excess QDs and resuspensions in HEPES buffer (10 mM, pH adjusted to 8.0 using 1 M NaOH).

#### 2.4.2 Covalent Route

Cross-linking agents *N*-(3-(dimethylamino)­propyl)-*N*′-ethylcarbodiimide hydrochloride (EDC) and *N*-hydroxysulfosuccinimide sodium salt (sulfo-NHS) were first added to a 1 μM QD655 stock solution at a 5:1 mM ratio. This mixture was gently shaken for 30 min to activate the QDs. Subsequently, the QDs were added to PEI-coated NRs in excess at a [1]:[12] loading ratio. The combined solution was incubated at 4 °C overnight, after which the pTA growth method detailed above was applied.

### 2.5 Nanoparticle Characterization

UV–vis spectroscopy was performed using an Agilent Cary 60 spectrophotometer with quartz cuvettes. ζ-Potential measurements were performed on a Malvern Panalytical Zetasizer Nano instrument using 500 μL of diluted samples in disposable microcuvettes. Prior to analysis, a zeta standard solution was measured with an acceptable range of 40 ± 5 mV. Bulk fluorescence spectroscopy was carried out on a Cary Eclipse fluorescence spectrometer with quartz cuvettes. An excitation wavelength of 435 nm was selected, and the excitation and emission bandwidths were adjusted to ±10 nm. The recording range was set from 455 to 750 nm with a data interval of 1 nm and a photomultiplier (PMT) voltage of 500 V for fluorescence analysis. Rayleigh-based nanoparticle tracking analysis (NTA) was performed on a Nanosight LM10 instrument. Sample solutions for analysis were further diluted in 0.2 μm pore filtered Milli-Q water. Videos were captured with a duration of 120 s and Nanoparticle tracking analysis (NTA) performed on software version 2.1. For scanning electron microscopy (SEM), nanoparticle images were obtained by using a FEI Quanta 250 FEG-ESEM field environmental SEM instrument operating in high vacuum mode. Transmission Electron Microscopy (TEM) images were acquired using a JEOL 1400FLASH at the School of Infection and Immunity department at the University of Glasgow.

### 2.6 Dual-Modal Imaging and Tracking for Single Nanoparticle Analysis

The dual-modal imaging and tracking system for single nanoparticle analysis is pictured in the Supporting Information (Figures S1 and S2) and applied for both single nanoparticle imaging when immobilized on glass coverslips and multimodal dynamic imaging of nanoparticles in suspension. This system features a customized Nikon LV100 upright microscope utilizing a Nikon 50× (NA = 0.55) ELWD BD objective in both cases. ***Single nanoparticle immobilization on glass coverslips***: PEI-coated glass coverslips were used to obtain dark-field and fluorescence images of immobilized single nanoparticles at a low surface coverage. High precision glass coverslips (22 × 22 mm^2^, 1.5) were prepared by washing with isopropanol and drying under nitrogen, followed by treatment in an oxygen plasma cleaner (Diener Femto low-pressure plasma system) for 5 min to create a hydrophilic, negatively charged surface. The coverslips were subsequently immersed in a branched PEI (1 mg mL^–1^) solution in aqueous 5 mM NaCl for 30 min, rinsed thoroughly with water and dried with a N_2_ stream to achieve a stable positively charged surface. Sample solutions (OD ∼ 1) were diluted 100-fold and approximately 100 μL was pipetted onto the coverslip surface. The solution was left to sit for at least 1 h in a humidity chamber to prevent drying-induced aggregation before gently rinsing and drying with a N_2_ stream. Reflected dark-field and fluorescence images of the same area were acquired using a Coolsnap HQ CCD camera and MetaMorph Version 6.3 software. For fluorescence imaging, the setup included a filter cube consisting of an exciter (Semrock FF01-435/40-25), dichroic (Semrock Di02-R514)/and emitter (Semrock FF01-650/60-25), allowing specific detection of QD655 with a single camera switching between dark-field and fluorescence. ***Dynamic dual-modal imaging of suspended particles***: A 3D-printed flow cell made of black acrylonitrile butadiene styrene (ABS) was designed featuring a single linear channel (ca. 30 mm length, 3 mm width, and 4 mm depth). To enable imaging, a glass coverslip was sealed over the top of the channel, plus glass windows were attached to each opposite end of the channel with inlet and outlet channel ports also drilled into the channel side walls (see Figure S2). A 488 nm beam (Melles-Griot Laser 43 series tunable argon-ion laser) is passed through a 488 nm narrow band-pass filter and directed through the channel via a 20 cm focal length PCX lens with beam steering applied so that the beam focal point spatially coincides with that of the 50× imaging objective lens.^13^ Scattered light collected from the sample solution is then distributed between two channels by using a 55:45 beam splitter integrated into the microscope housing. Rayleigh scattered light from one channel was imaged with an Andor 885 EMCCD, which was attached to an Andor Shamrock 303i monochromator with both grating and mirror options. Fluorescence images were captured by using an Andor 897 EMCCD equipped with a bandpass filter (Semrock FF01-650/60). Both cameras have very similar chip sizes, with *x* and *y* lateral offset adjustments made to enable both cameras to cover the same field of view. The 1004 × 1002 pixel EMCCD 885 was binned at 2 × 2 compared to the 512 × 512 pixel EMCCD 897, which was operated without binning. Other parameters such as integration time, shift speed, and gain settings were optimized independently for the two very different signal intensities in each detection channel. Synchronized kinetic videos were acquired using Andor SOLIS Version: 4.30 at a typical frame rate of ∼10 Hz with one camera triggering the other. The time delay between each camera image acquisition is on the order of microseconds and much shorter than the diffusional speed of the nanoparticles to enable co-localization between both imaging channels. ImageJ was used for the processing and analyzing of all imaging data. Additional details are also provided in the Supporting Information (Figure S3).

## 3 Results and Discussion

### 3.1 Design of Custom Functionalized AuNR–QDs

To enable the goal of creating a stable colloidal AuNR-QD nanotag scaffold that is optically bright and can be readily functionalized for further application development, we explored the two different routes shown in Figure 1. In both cases, the synthesis involves several key steps for achieving stabilized single nanoparticle assemblies suitable for dynamic dual-modal imaging and tracking in suspension. First, CTAB-stabilized AuNRs were prepared by adapting a previously reported hydroquinone seed-mediated growth approach, favored for their tunable LSPR and enhanced scattering for optical imaging applications.^47^ The characteristic rod shape and particle uniformity resulting from this synthetic procedure are pictured in Figure 2, with additional images provided in the Supporting Information (Figures S4 and S5).

**Figure 1:**
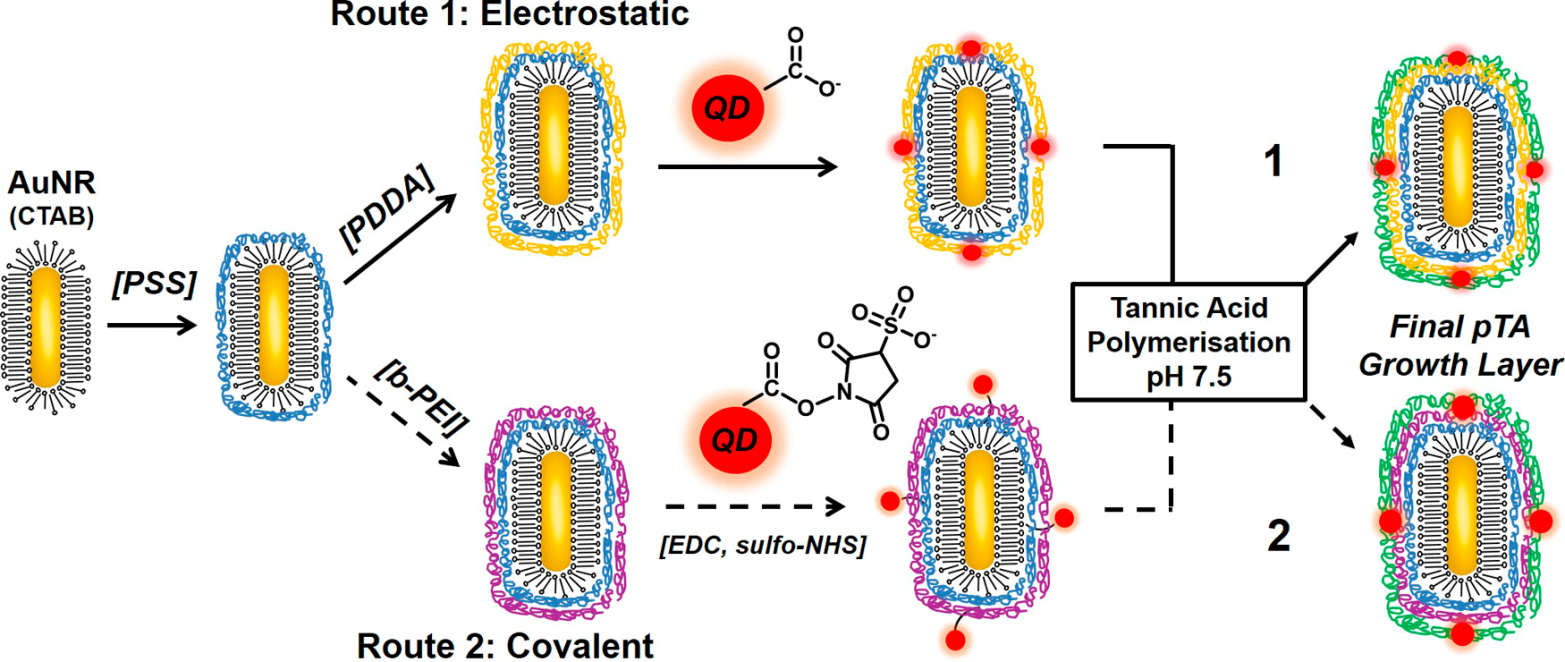
Schematic overview of the two synthetic routes explored for functionalized AuNR–QDs as optical nanotags for dual-modal single nanoparticle imaging and tracking. Both routes feature sequential self-assembled polyelectrolyte coatings and an outer pTA layer. The electrostatic route 1 uses a different cationic polyelectrolyte (PDDA) in comparison to the covalent route (branched-PEI), where additional EDC/NHSS covalent coupling agents were utilized for QD conjugation. In both cases, QDs emitting at 655 nm were used.

**Figure 2:**
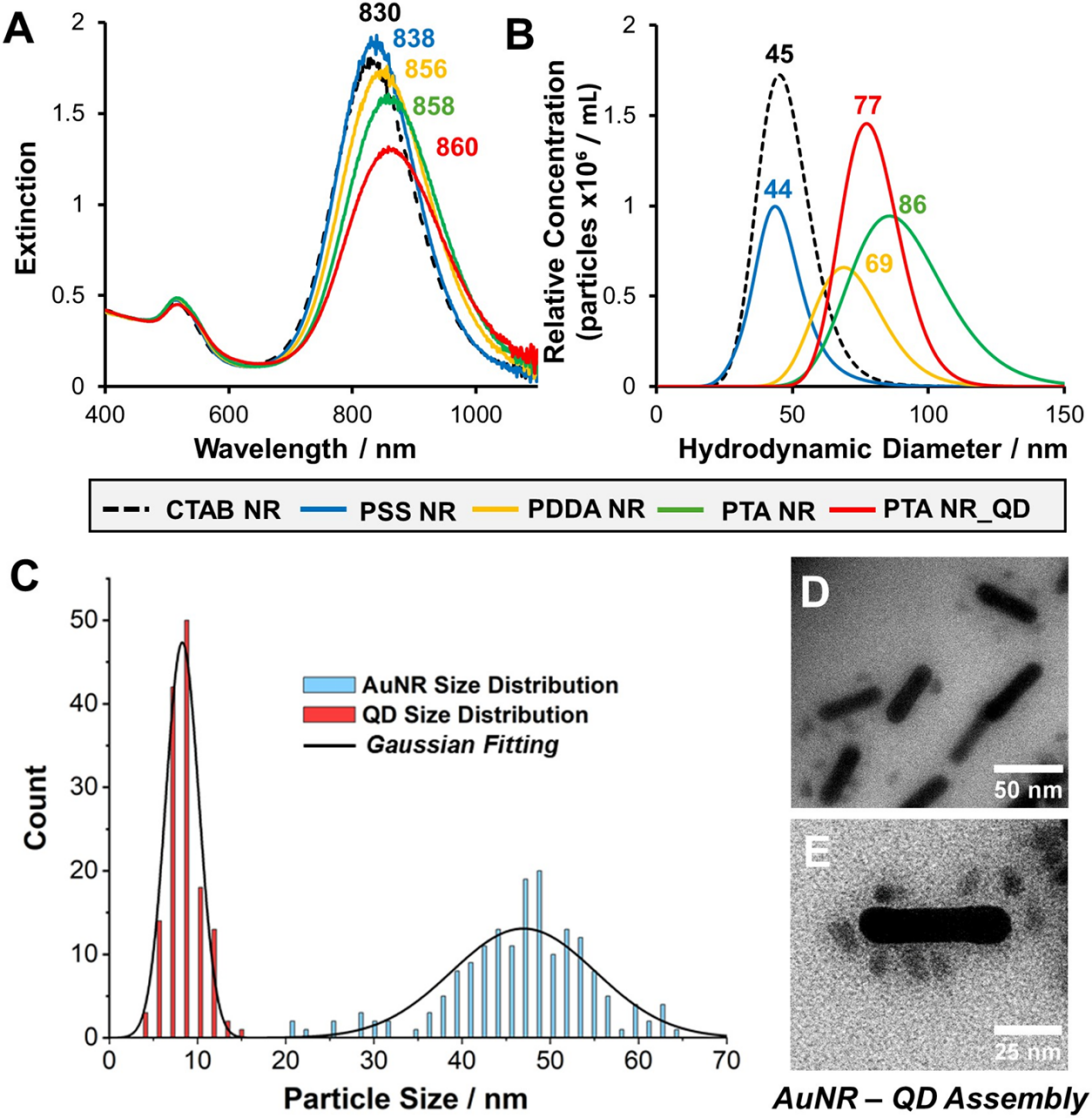
Characterization of AuNRs prepared via electrostatic route 1. (A) UV–vis spectroscopy, with spectral intensities normalized at 450 nm to aid comparison. The LSPR λ_max_ is also highlighted. (B) NTA characterization with the peak positions included. In both cases, the outer surface functionalized layer is indicated by a different curve color. (C) Size distribution of AuNR longitudinal length and QD diameter obtained from TEM analysis with representative images of the AuNR-QD conjugates shown in (D) scale bar = 50 nm and (E) scale bar = 25 nm.

Next, ultrathin polyelectrolyte coatings were deposited on the nanorod surface via the well-established electrostatic layer-by-layer (LbL) approach.^50^ Following the deposition of an initial anionic PSS layer and the removal of excess CTAB, the cationic layers of either PDDA (Route 1) or branched-PEI (Route 2) were self-assembled. Most of the development work reported here was initially performed via Route 1 where the self-assembly of carboxyl-terminated QD655’s was controlled via electrostatic interactions, with the PDDA coating providing a strongly charged cationic surface that is stable over a wide pH range.^51,52^ The Route 2 alternative of covalent coupling via EDC/sulfo-NHS between available surface amine groups provided by b-PEI and surface carboxyl groups on the QD was then comparatively assessed.

In both methods, a poly­(tannic acid), pTA, outer layer was grown to achieve QD encapsulation within the nanostructure as well as improve the stability of the assembly. The choice of pTA was based on a number of reasons: the mild alkaline conditions and relatively short reaction times (90 min) enable a controlled oxidative polymerization reaction resulting in a layer thickness of around 10–20 nm. Also, measurements were performed to help establish that the pTA layer formation can occur on both positively and negatively charged surfaces. While this report focuses on pTA growth on a positively charged PDDA surface, the Supporting Information (Figure S6) shows pTA growth on PSS-coated nanorods. Spectral analysis of these functionalized nanorods shows similar changes, further supporting successful pTA growth on negatively charged surfaces. As discussed later, the formation of thin pTA layers only partially quenches the QD655 emission. In contrast, preliminary measurements involving polydopamine (PDA) formation indicated complete QD quenching and this typically also involves longer polymer layer growth times.^53,54^ Finally, the stability of the polymer matrices formed by pTA layers combined with providing a functionalized surface that can be integrated into a wide range of applications is highly desirable for application purposes.^49,55^

The larger nanoassemblies promote slower diffusion speeds in suspension compared with individual QDs, as explained by the Stokes–Einstein equation. This size dependence has been observed across various nanoparticle systems, where the increased particle size correlates with improved optical imaging contrast. Such enhancements can support the visualization of single nanoparticles in dynamic imaging and tracking applications.^56,57^ Additionally, the controlled separation distance between QDs and NRs, achieved with ultrathin polyelectrolyte layers, minimizes fluorescence quenching, thereby promoting enhanced optical imaging.^58^

### 3.2 Bulk Characterization of AuNR-QD Conjugates

Each step of the AuNR-QD assembly process was monitored via a series of characterization techniques. For samples prepared via the electrostatic Route 1, the corresponding UV–Vis spectra are shown in Figure 2 alongside nanoparticle tracking analysis (NTA) and representative TEM images with further optimization discussed later in Figure 3. In addition, a similar set of measurements were performed for monitoring covalent Route 2, whereby the optimal AuNR-QD concentration ratio identified in Route 1 produced similar results (see Figure S7).

**Figure 3:**
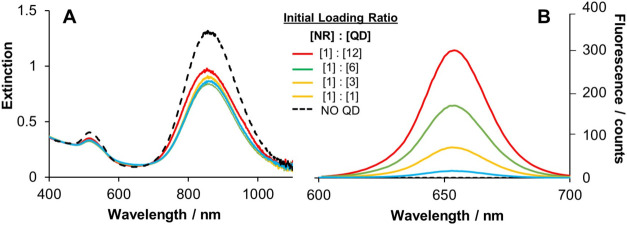
Monitoring of AuNR-QD assemblies prepared at various [NR]/[QD] concentration loading ratios via electrostatic Route 1 with the initial NR concentration fixed at 0.3 nM for each sample. The corresponding (A) extinction and (B) bulk fluorescence spectra are shown. Prior to analysis, each sample was pTA wrapped, washed repeatedly and the particle concentrations normalized with respect to the extinction at 450 nm to aid comparison. QD emission was acquired at an excitation of 435 nm.

The spectral data in Figure 2A shows a red shift from 830 to 838 nm in the nanorod LSPR λ_max_ upon PSS coating of the stock NR solution with successful wrapping also supported by the measured ζ-potential shifting from +32.1(±7.6) mV for CTAB-AuNRs to −26.9(±7.3) mV for PSS-coated rods.^39^ Successive λ_max_ red shifts to 856 and 860 nm were observed for the PDDA and the QD/pTA coatings, respectively. Changes in the nanorod surface ζ-potential from +21.9(±8.7) mV (PDDA NR) to −29.9(±9.2) mV (pTA NR) also supports effective coating and colloidal stability. A comparison of pTA coating both in the presence and absence of adsorbed QDs is also included. As expected, the ζ-potential of the pTA NRs (−29.9 mV) is similar to that of the pTA NR–QD’s. However, there is a trend of increased damping of the NR LSPR extinction maximum for each additional layer with the final pTA NR-QD conjugate typically 0.6 times the intensity of the original PSS-coated NRs. This is associated with the structural properties of the pTA layer, which features increasing electronic conjugation with thickness as well as the refractive index properties of the inorganic QDs.^59–61^ In addition, FTIR measurements were performed to further confirm the addition of each polymer layer (Figure S8).

A key concern for the electrostatic assembly route is to ensure that there is no uncontrolled formation of larger nanoparticle assemblies involving multiple nanorod templates. Systematic nanoparticle tracking analysis (NTA) measurements (Figure 2B) indicated good control over the assembly process, with very few large aggregates observed in repeated videos. It should be noted that the hydrodynamic diameters reported cannot be directly attributed as absolute values since the calculated diffusion constants and associated particle diameters are based on spherical systems compared to the anisotropic rod shapes involved here.^62^ However, it is still instructive to note the relative trends and changes in the size distribution upon the addition of each layer in the assembly process.^63,64^

The TEM images in Figure 2D,E and in the Supporting Information (Figure S5) provide further insight into the formation of AuNR-QD assemblies in a colloidal solution. These images are for a sample prepared at a [1]:[12] [NR/QD] concentration ratio (discussed in the next section). Further TEM image analysis revealed that there are no significantly large NR aggregates present (i.e., >100 nm). However, it is worth highlighting here that the image contrast of the smaller QD’s versus the larger metallic nanorods means that it is difficult to identify a QD when it is spatially overlapping with a NR. As a result, we found that only ∼66% of the NR’s were visually confirmed as conjugated to at least one or more QD, while the optical analysis results of the same sample described later support a much higher percentage. Also, there are several QD cluster formations observed in close proximity to nanorod surfaces, which may be due to aggregation in the original supplier stock or interactions with the growing pTA layer and during the centrifugation/wash cycles required during the sample preparation to remove excess QD’s. Additionally, drying-induced aggregation may occur when preparing the TEM substrate. The majority of QD aggregates observed are ∼40 nm in size, with a few reaching up to 120 nm. Further characterization of individual QDs versus clustered QDs, formed through tannic acid polymerization, shows an increase in particle size due to the presence of pTA (see Table S1). Despite the presence of QD aggregates, as reported by DLS measurements (Table S2), their size remains within a range that is still effective for dynamic imaging analysis, comparable in size to singular AuNR–QD assemblies.

#### 3.2.1 Optimization of AuNR-QD Assemblies

As part of the Route 1 development, the concentration ratio of NRs and QDs (i.e., [AuNR]/[QD] mixed together during the assembly process) was also investigated. Figure 3 shows the corresponding UV–vis and bulk fluorescence spectra when this preparation concentration ratio is varied from [1]:[1] to [1]:[12]. In each case, the nanorod concentration was fixed at 0.3 nM and the QD concentration was adjusted accordingly before completing the pTA wrapping and sample cleanup to remove excess QDs. After AuNR-QD concentration normalization based on the AuNR extinction at 450 nm, the corresponding fluorescence spectra were acquired and compared. The LSPR extinction and QD emission peak details are reported in the Supporting Information (Table S3) along with data (Figure S9) taken for QDs only (without AuNRs) for bulk fluorescence. At much higher [AuNR]/[QD] ratios, an excess of QD’s results in more extensive aggregation, causing colloidal solution destabilization and excessive QD loading in the preparation stage. While higher concentration ratios during preparation can improve the signal-to-noise for single nanoparticle imaging analysis, this parameter must be carefully optimized to maintain the stability and functionality of the AuNR-QD assemblies.

The spectral comparison of conjugates in Figure 3A shows that as the [AuNR]/[QD] ratio was increased, the longitudinal LSPR peak intensity was further damped, while the position of the λ_max_ changed only slightly. For the QD fluorescence measured at the highest initial loading ratio, [1]:[12], the intensity was typically more than 10-fold higher than the lowest particle concentration sample solution, [1]:[1], with the emission λ_max_ red-shifting only ∼2 nm in the presence of rods compared to QDs only. The impact of pTA encapsulation on the QD emission was also investigated. Figure S10 compares a separate data series in which both extinction and fluorescence spectra were compared before and after the final pTA washing step for each of the concentration loading ratios. Analysis of the QD emission shows a further λ_max_ red shift of ∼2 nm occurring in each case due to pTA wrapping. In addition, the bulk emission intensity decreased by ∼50%. Accordingly, the NR LSPR λ_max_ also red shifts ∼12 to 13 nm and is damped by ∼20% upon pTA coating only. These comparative measurements were performed after repeated washing steps to remove excess pTA and QD’s and using the extinction spectra to attain comparative NR-QD concentrations.

These spectral measurements support that increasing the concentration loading ratio results in an increase in the average fractional surface coverage of QDs surrounding each nanorod, and this is discussed further in the next section. However, at higher QD concentrations, additional washing cycles were required to remove excess QDs from the colloidal solution. The Supporting Information (Figure S11) includes fluorescence spectra for the supernatants of each loading ratio after three wash cycles, demonstrating that excess QDs were effectively removed at higher loading ratios. The fluorescence emission of the AuNR–QDs was observed to remain stable for >2 months before significant degradation, as shown in Figure S12. A small lowering in colloidal concentration as indicated by UV–vis characterization may also have contributed to the gradual lowering in fluorescence intensity of the stored samples that is observed at ∼3 months storage.

Additional photoluminescence lifetime measurements are presented in Figure S13, which compares the optimized AuNR-QD assemblies with a colloidal solution of QD655’s only. This data clearly highlights a much shorter decay lifetime for the AuNR-QD’s. The PL emission curves were fitted to a three-exponential decay function, which determined an amplitude average lifetime *<*τ*>*_amp_ of 6.9 (±0.2) nS for the AuNR-QD’s versus 30.1 (±0.7) nS for the QD’s. A significantly faster average decay lifetime is expected for optical coupling between QD’s and plasmonic nanoparticles.^32,65^ The number of noncoupled QD’s in the AuNR-QD colloid is minimal and will also contribute to the contrast between both measurements. In this study, the focus of our approach is to obtain optically bright assemblies suitable for dual imaging and tracking, and we have not attempted to also quantify the relative enhancement (or quenching) of the QD emission. This requires a different optical configuration and the interplay between PL enhancement and quenching for plasmonic-QD systems has been summarized elsewhere.^22^

The formation of AuNR-QD assemblies in suspension can also be achieved using the covalent coupling route, whereby equally effective polyelectrolyte nanorod coatings and stabilized nanostructures were formed. A summarized data set in the Supporting Information (see Figure S7) presents the optimal AuNR-QD loading ratio ([1]:[12]) studied in the covalent method, Route 2, which highlights comparable results at this concentration.

### 3.3 Single Nanoparticle Immobilization of AuNR-QD Assemblies

Prior to performing dual imaging monitoring of individual nanoassemblies in suspension, optical characterization measurements were first performed by immobilizing individual nanostructures at low surface coverage on glass coverslips. Figure 4 shows representative dark-field and fluorescence images of the same substrate area for a sample prepared with a [AuNR]/[QD] ratio of 1:12. The coverslips were functionalized and coated following a procedure that promoted particle adsorption and also minimized drying-induced aggregation.

**Figure 4:**
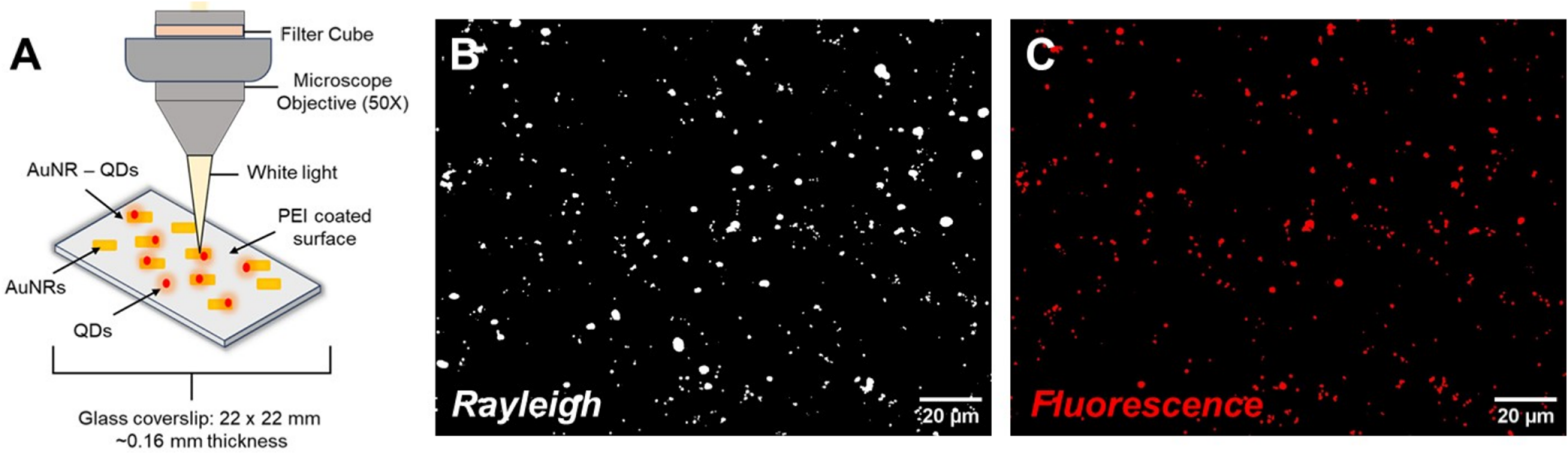
Dual imaging of AuNR-QD assemblies immobilized on a glass coverslip (A) with the same area imaged by (B) dark-field/Rayleigh scattering and (C) QD fluorescence. The samples were prepared at a [AuNR]/[QD] concentration ratio of [1]:[12]. Scale bar = 20 μm for both images.

The direct comparison of both imaging modalities in Figure 4 provides insight into the AuNR-QD conjugation efficiency, which is defined by the yield, consistency, and overall stability of the nanoassemblies. Co-localization between dark-field and fluorescence images provides evidence of AuNR-QD assembly as Rayleigh scattering is enhanced by the AuNRs while no signal is observed from isolated QDs under the same acquisition conditions. Based on integration times and separate analysis of QDs only, the fluorescent acquisition conditions are sensitive enough to provide confidence that at least one or more QDs attached to an AuNR would be detected in the images. Additionally, no signal in the red fluorescence channel is observed from AuNRs in the absence of QDs (Figure S14).

A series of dual images across 5 different substrate areas were acquired for each of the [AuNR]/[QD] loading ratios and analyzed (see Figure S15 and Table S5). These results show a clear trend with the AuNR-QD yield increasing from 13 to 93% as the loading ratio is varied from [1]:[1] to [1]:[12]. While the focus of our approach was to determine if there was at least one or more QD’s attached to each NR, it was considered if further insight can be obtained on the average number of QD’s attached to each NR from the DF/Fluo imaging data. For the [1]:[12] ratio, looking at the intensity distribution of particle maxima in the DF and fluorescence images for both QD’s only and QD-NRs acquired under the same imaging acquisition conditions, a threshold pixel intensity value was set. Based on this, we estimated that at least ∼45% of the NR-QD’s have around 1–2 QD’s attached per NR. There is some uncertainty with this approach, for example, there could be more than one NR-QD assembly within a diffraction-limited spot and we have thus focused on the lower scattering intensity particles acquired across a series of sample images for this estimate. An advantage of this dual imaging approach is that larger numbers of assemblies can be readily analyzed compared to TEM imaging and with a higher contrast also. However, one disadvantage of static dark-field imaging is that a small percentage of image features will be due to defects in the coverslip glass and other unidentified point scatterers, which are not immobilized nanoparticles. Consequently, the absolute yields reported using this approach can be expected to be a little higher than that measured.

### 3.4 Dynamic Dual-Modal Imaging of AuNR–QD Assemblies in Suspension

To further improve the characterization of the AuNR–QD assemblies and enable application opportunities based on high-throughput imaging and the tracking of individual assemblies, a novel dual imaging approach was developed. While nanoparticle tracking analysis (NTA) of individual particles is now an established characterization technique,^63^ which includes the alternative switching between Rayleigh and fluorescence in a single channel,^66,67^ real-time dual imaging is needed for more complex assemblies and this has not been explored to any significant extent.^7^ Figure 5 shows the optical setup and individual frames from synchronized videos captured simultaneously in the Rayleigh and fluorescence scattering imaging channels. An advantage of the dual camera approach is that parameters such as the gain and integration time can be independently adjusted to compensate for the very different acquisition settings required for each imaging modality. In contrast, an approach involving splitting a single image through separate filtered channels onto the same camera chip reduces the available field of view and requires the same camera acquisition settings to be sufficient for both channels.^21,68^

**Figure 5:**
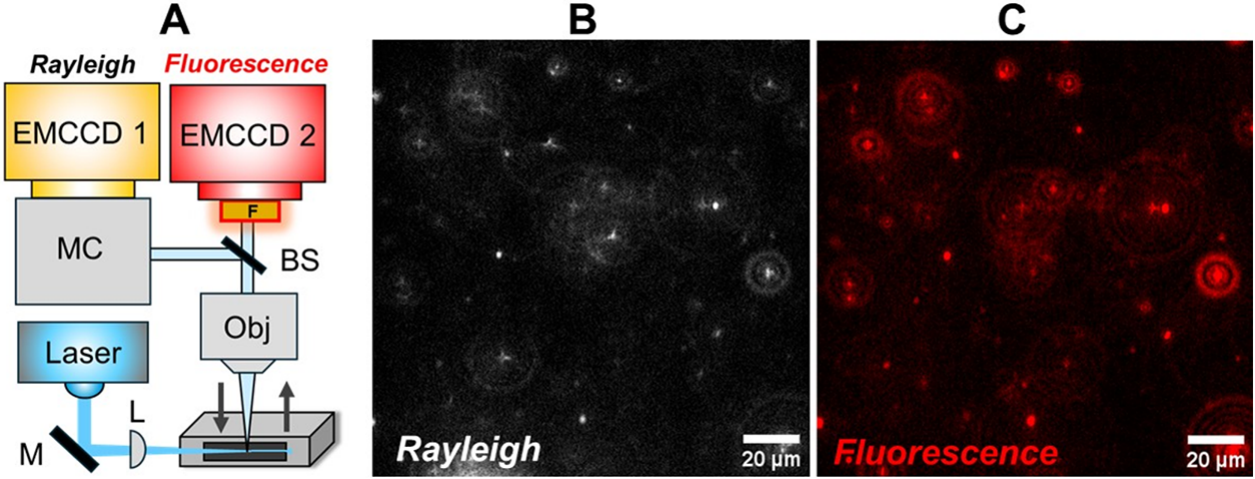
Real-time dual imaging and dynamic tracking of individual AuNR-QD assemblies moving freely in suspension. The sample was prepared with a [1]:[12] loading ratio. A simplified optical setup is shown in part (A) along with time-synchronized original video frames from separate part (B) Rayleigh and part (C) fluorescence channels. Scale bar = 20 μm in both images. M – Mirror, MC – Imaging Monochromator, L – Lens, Obj – Microscope Objective, BS – Beam Splitter, F – Filter.

Prior to imaging analysis, calibrations were performed to determine the *x* and *y* lateral offsets between both channels, and the optical path lengths were also adjusted to ensure both cameras were focused on the same image plane. It was also necessary to compensate for a small difference in image magnification between each optical path with the Rayleigh channel also passing through an imaging monochromator (see Figure 5A). See Figure S3 and accompanying description for more details on the dual-imaging setup.

The imaging data in Figure 5 are for AuNR–QD samples prepared at the highest [1]:[12] concentration loading ratio with representative frames of the Rayleigh and fluorescence scattering captured simultaneously shown in Figure 5B,C, respectively. Example videos for both channels are also provided in the Supporting Information (Video S1). Also provided in the Supporting Information are images for the AuNR-QD assembly prepared at lower ratios and captured under the same acquisition settings. Co-localization analysis between the Rayleigh and fluorescence videos was then performed with data from both channels compared on a frame-by-frame basis.

The comparative analysis of the Rayleigh and fluorescence scattering videos for the highest [1]:[12] concentration loading ratio resulted in a yield of 97% where it can be assumed that at least one QD is conjugated to a AuNR core, with further video analysis summarized in Table S6. As expected, this yield is slightly higher than the 93% reported earlier for the immobilized sample analysis (Figure 4). The larger nanoassemblies enhance both the Rayleigh scattering and fluorescence brightness when compared to individual QDs, enabling high signal-to-noise ratios and slower diffusion speeds, which is favorable for dynamic single nanoparticle imaging analysis. In comparison, the lowest concentration loading ratio of [1]:[1] resulted in a yield of 52% under the same dynamic conditions (as shown in the videos referenced in Table S7). Although this yield is greater than the 13% reported for the immobilized sample analysis (Figure S15 and Table S5), it remains notably lower compared to the [1]:[12] nanoassembly. This is because a larger number of QDs are available to conjugate with the AuNR core at higher loading ratios, leading to an increase in the number of successful conjugation events. Additionally, the reduced scattering and fluorescence brightness at lower concentration loading ratios suggest that fewer QDs attach to individual rods, resulting in weaker signals in dynamic imaging. In contrast, higher QD loading ratios improve conjugation efficiency and signal-to-noise ratios while maintaining high stability in colloidal solution. The difference between 13 and 52% is likely attributed to the immobilization techniques inability to detect weaker signals effectively. CCD cameras used for single nanoparticle immobilization lack the sensitivity and amplification of EMCCD cameras in dynamic dual-modal imaging, where real-time tracking enhances the detection of successful single particle conjugation events at lower QD loading ratios. Also, further insight into other aspects such as the number of QD’s attached to a single NR diffusing randomly in and out of the focal plane will require further development of both the image analysis tools and optimization of video acquisition parameters.

Dynamic dual-imaging data for AuNR-QD assemblies prepared via the covalent coupling route at [1]:[12], under the same analysis conditions, can be found in the Supporting Information (Video S2). It was found that AuNR–QD assemblies prepared via covalent coupling to be just as effective in terms of conjugation yields as the electrostatic route that has been predominantly discussed throughout this article.

Real-time videos of individual QDs in the Supporting Information (Video S3) serve as a control, highlighting the challenges in dynamic imaging analysis compared to the significantly improved performance of larger, singular nanostructures such as the AuNR-QD assemblies. The slower diffusion rate of AuNR-QDs (∼3 μm^2^ s^–1^) compared to individual QD (∼25 μm^2^ s^–1^) makes them more distinguishable and allows for the collection of significantly higher optical signals, enhancing signal-to-noise ratios and improving the visualization of single AuNR-QD assemblies. During the assembly process, some QD aggregates are inevitably produced. These aggregates, typically small (around 40 nm as observed in the TEM images provided in the Supporting Information, Figure S5) remain difficult to detect, resulting in poor imaging contrast and reduced signal-to-noise ratios in dynamic imaging analysis, as demonstrated in the Supporting Videos (Video S4), where clustered QDs were formed through tannic acid polymerization and high-speed centrifugation. The small clusters continue to exhibit faster diffusion rates (6–25 μm^2^ s^–1^) compared to those of AuNR-QD assemblies. Further data, including the size, ζ-potentials, and single particle immobilization images of individual and clustered QDs are provided in the Supporting Information (Table S2 and Figure S16) to emphasize the changes in particle characteristics and behavior during the formation of AuNR-QD assemblies.

Larger QD aggregates, stabilized by the pTA layer, can reach >120 nm in size (observed in TEM analysis reported in Figure S5) and exhibit enhanced Rayleigh scattering (see Figure S16), similar to AuNR-QD assemblies, due to their slower diffusion speeds (2 μm^2^ s^–1^). Although these larger aggregates are relatively rare and may not be detected in the NTA data, they could still prove to be advantageous for dynamic imaging analysis, potentially serving as effective nanotags alongside AuNR-QD assemblies in colloidal solutions.

Comparing the dual imaging approach demonstrated here with other nanoparticle imaging systems, a key technical aspect is the two separate optical paths where a combination of filters and wide-field EMCCD’s can be adjusted independently to enable colocalized measurements of the same diffusing nanoparticle assembly. This is essential because the photon detection sensitivity required for the Rayleigh and fluorescence modalities is different by several orders of magnitude, and switching between modalities on a single channel is not feasible for monitoring the same suspended nanoparticle. An example of Rayleigh and fluorescence/SERS dual channel imaging of nanoparticles in suspension has been previously reported with the microscope image split into two optical paths and then focused onto separate halves of the same camera EMCCD.^19^ However, this significantly limits the measurement dynamic range across both modalities and reduces the field of view throughput. Other techniques such as interferometric scattering microscopy^69^ and multicolor TIRF microscopy^70^ have been powerfully demonstrated for 3D tracking, however, both of these are still limited to a single imaging modality and are restricted to a short optical working distance beyond the glass coverslip and incident microscope objective. In our case, the imaging depth and resolution depend on the use of objectives with long working distances of several millimeters combined with optically bright nanoparticles whose concentrations are sufficiently diluted to minimize image background contributions while still achieving high particle throughput.

## 4 Conclusions

In this work, we optimized an approach for the preparation of functionalized gold nanorod-QD nanoassemblies, which was also combined with the development of a dual-modal imaging approach for characterizing individual assemblies in suspension. Both electrostatic and covalent routes were successfully demonstrated for conjugation of QD’s to NR’s with the formation of a poly­(tannic acid) outer layer imparting both long-term stability of the NR-QD conjugate and providing an established platform for further covalent molecular functionalization. Furthermore, the introduction of a novel approach for correlated Rayleigh scattering and fluorescence imaging of freely diffusing individual nanoparticles offers several advantages over static optical and electron microscopy analyses of individual particles immobilized on a surface. These include higher-throughput analysis and minimizing artifacts such as drying-induced aggregation. Attaining greater confidence in the AuNR and QD conjugation rates also helps to optimize assembly parameters such as the relative concentrations of both particles. In the near future, the combination of dynamic multimodal dual imaging, higher single nanoassembly throughput plus further development in real-time image analytics will be important for the future development of nanoparticle-based analytical assays and working with more complex colloidal mixtures and environments.

## Supplementary Material











## Notes

Images of experimental setup (Figures S1–S3); TEM images (Figures S4 and S5); UV–vis characterization of functionalized NRs (Figure S6); characterization data of QD-NR’s prepared via Route 2 (Figure S7); FTIR of coated NPs (Figure S8); fluorescence spectra monitoring the optimization of NR-QD assembly (Figures S9–S11); stability study (Figure S12); photoluminescence lifetime measurements (Figure S13); comparative dark-field and fluorescence imaging of surface-immobilized NPs on coverslips (Figures S14–S16); summarizing particle characterization data of AuNR’s (Table S1), QD’s (Table S2), and NR-QD assemblies at different preparation conditions (Tables S3 and S4); analysis of dual imaging measurements on glass coverslips (Table S5) and in suspension (Tables S6 and S7) (PDF)
Representative dual modal videos of NR-QD assemblies prepared via Route 1 (Video S1) (AVI)
Route 2 (Video S2) (AVI)
Individual QD’s only (Video S3) (AVI)
QD aggregates (Video S4) (AVI)
