# Supplementary material for: Robust Development of Gold Nanorod-Quantum Dot Assemblies for Dynamic Dual-Modal Single Nanoparticle Imaging and Tracking Applications
Source: ACS Appl Nano Mater. 2025 Dec 8;8(50):23832–42. doi: 10.1021/acsanm.5c03231 (PMC12723642; doi:10.1021/acsanm.5c03231)
Supplement: Supplementary file 1 [file an5c03231_si_001.pdf]

<u>Supporting Information for:</u>

Robust Development of Gold Nanorod-Quantum Dot Assemblies for Dynamic Dual-Modal Single Nanoparticle Imaging and Tracking Applications

Hannah L. Taysum, Aryanne L. Finnie, Aimee McKay and Alastair W. Wark*

Technology and Innovation Centre, Dept. of Pure & Applied Chemistry, University of Strathclyde, 99 George St, Glasgow, UK, G1 1RD

***Corresponding author:** *Email: alastair.wark@strath.ac.uk, ORCID:* 0000-0001-8736-7566

Table of Contents

Supporting Figures 3
Supporting Tables 20
Supporting Videos 24

Supporting Figures

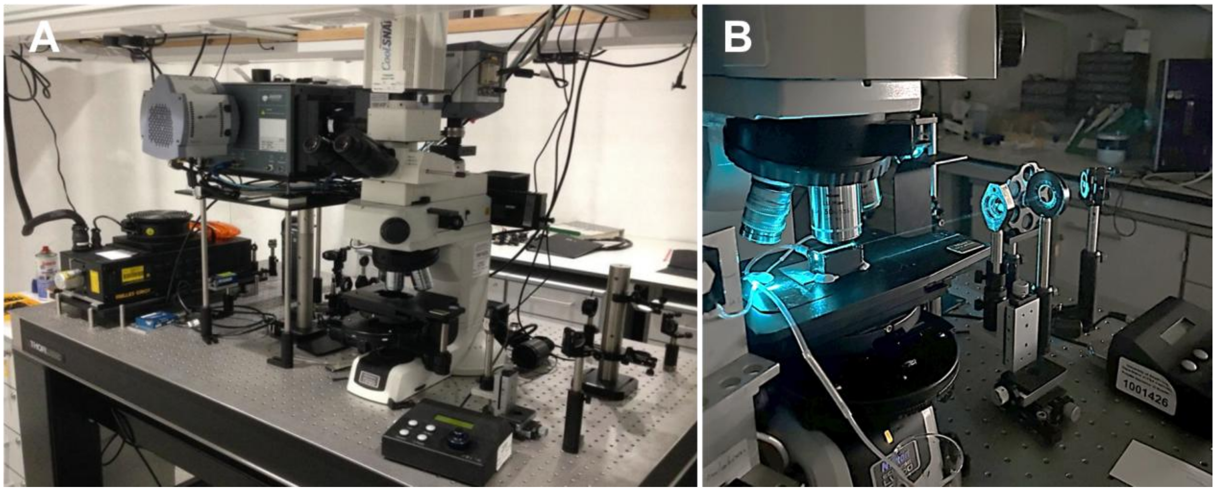

Figure S1. Experimental set–up of the dual modal imaging and tracking system for higher throughput single nanoparticle analysis in suspension. Pictured in A is the microscope set-up equipped with two EMCCD cameras, one of which is attached to a monochromator. Pictured in B is the argon-ion laser source at 488 nm excitation entering the 3D printed flow cell via a 20 cm focal length PCX lens. A beam block at the exit side of the channel is usually in place also.

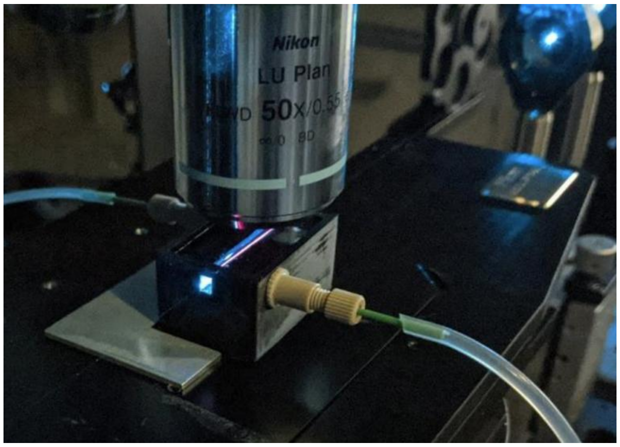

Figure S2. Image of the 3D-printed flow celled with the argon-ion laser passing through the chamber. A glass coverslip was sealed over the top of the channel to allow for imaging, pictured using a 50X ELWD microscope objective. There are glass windows at each opposite end of the channel along with inlet and outlet ports on the channel side walls to allow solution to be pumped through the linear channel.

Additional Dual-Imaging setup details

To achieve simultaneous dual modal imaging on the system described, EMCCD 1 (Andor 885) and EMCCD 2 (Andor 897) cameras were carefully aligned and calibrated along the x, y and z-axes prior to imaging analysis. Camera alignment was required due to the differences in sensor resolution, physical chip size and optical path geometry between the two detectors. In the optical setup, the Andor 885 camera was fixed in position and served as a reference channel for alignment. The Andor 897 camera was mounted on a precision X-Y translation stage fitted with a fine-focus threaded tube spacer, allowing incremental position adjustments relative to the optical axis of the 885. The translation stage enabled lateral (x-y) alignment to ensure that both detectors captured overlapping regions of interest within the field of view, while the threaded spacer provided fine axis (focal plane) correction to match the imaging depth of the reference channel.

The Andor 897, despite having a lower pixel density (512x512 pixels; 16x16 μm) compared with the Andor 885 (1004 x 1002 pixels; 8x8 μm), has a similar chip area. However, an additional difference arises from the optical pathway where the image directed through the monochromator port experiences a slight change in magnification, as the optical system is not 1:1 at the entrance and exit ports. Inside the monochromator, the grating is replaced by a flat mirror, but the optical expansion and reduction process via confocal mirrors still introduces a minor magnification variance. To quantify these differences and ensure accurate channel colocalization, a micron-scale calibration grid was used under brightfield illumination with the 50X LWD microscope objective. Bright field images from both cameras were captured and analysed in ImageJ to establish a pixel-to-micron conversion. This yielded 10 μm = 72 pixels for EMCCD 1 (Andor 885) and 36 pixels for EMCCD 2 (Andor 897). As described in the experimental section of the article, the Andor 885 videos were subsequently binned at 2x2 pixels while the Andor 897 was not binned. Following camera alignment and image acquisition, the X and Y signal offset between the two image frames were calculated and the EMCCD 2 image was cropped to match the field of view for EMCCD 1 then rescaled to 512x512 pixels. EMCCD 1 image processing only required rescaling, for which the image was binned 2x2 (502x501 pixels), then similarly rescaled to 512x512 pixels. Verification of accurate image calibration was achieved by remeasuring the calibration grid squares. Following careful camera alignment, EMCCD 2 camera was securely clamped to prevent a mechanical shift or misalignment during subsequent imaging. The two imaging channels were merged to validate successful signal offset correction. The alignment of the micron scale square features on the calibration grid across both channels confirmed minimal spatial discrepancy.

Image Acquisition Settings: Image acquisition was performed using Andor Solis (Version 4.30) with the more sensitive 897 EMCCD used as the fluorescence channel and which also externally triggered the 885 image acquisition during a kinetic data series to enable time synchronization. The acquisition parameters were optimized and maintained throughout the imaging analysis to ensure reliable data collection. For example, the EM Gain (typically 25 for 885; 100 for 897) and

integration times (0.07s for 885; 0.06s for 897) were adjusted to amplify weak fluorescence signals without significantly increasing the exposure time difference between the two cameras. Also, the spooling mode was enabled to automatically save acquired image sequences in a sif file format to a designated folder which facilitated ease of data collection and handling. EMCCD 1 was operated in external trigger mode, supporting simultaneous acquisition upon internally triggering EMCCD 2 which allowed for automated frame by frame time synchronization between the two channels. The two cameras readout rate, pre-amplifier gain and shift speed were individually optimized to enhance signal-to-noise ratios.

Figure S3 compares average cross-sectional line profiles from images of multiple individual NR-QD's and QD's taken from selected frames provided in the supporting information videos (S1 and S3 respectively).

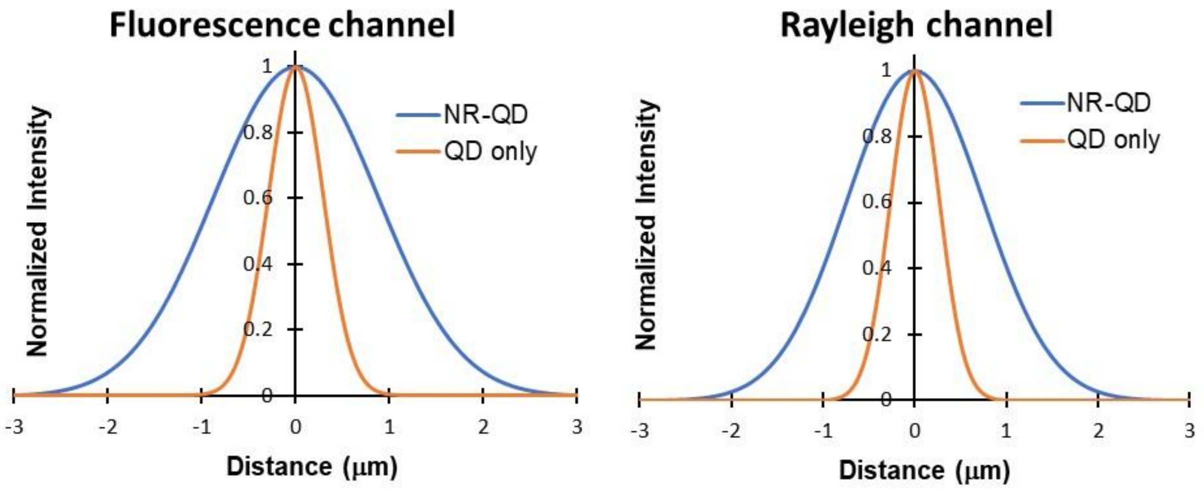

Figure S3. Normalized cross-section line profiles of individual NR-QD's and QD's taken from the analysis of multiple (in focus) particles within different image frames featured in the supporting information videos (S1 and S3, respectively).

The FWHM's from the linewidth analysis of multiple single particles in a dynamic video frame alongside an average of the S/N for the particle image intensity versus the background is summarized below.

**Sample Type**	**Channel**	**Mean S/N**	**Typical FWHM (μm)**
NR–QD Assemblies	Rayleigh (885)	25.3	1.75
NR–QD Assemblies	Fluorescence (897)	11.6	2.16
Individual QDs	Rayleigh (885)	7.4	0.69
Individual QDs	Fluorescence (897)	4.1	0.63

The S/N was calculated from intensity normalized images (between 0 and 1) and the mean background intensity $\sigma_{background}$ determined. Generally, the Rayleigh channels have a consistently lower background pixel intensity variation ($\sigma \approx 0.04$–0.10) providing higher particle signal contrast. The fluorescence channels involve measurements at higher gain settings with more detector noise and uneven background signal ($\sigma \approx 0.15$–0.25), explaining the lower S/N. In general, the larger and slower diffusing NR-QD's result in a greater point spread function as well as a higher S/N. The imaging performance could be improved through the use of a higher NA objective lens. However, the optical geometry of the setup requires an objective lens working distance of at least 2-3 mm into the flow channel.

Image Colocalization analysis: Videos captured during dual dynamic imaging and tracking were preprocessed using Image J software. The acquired .sif files were opened on Image J. The image format was converted from 32-bit to 8-bit to ensure compatibility for further processing. Brightness and contrast were adjusted manually. Typically, background subtraction was applied and, if necessary, image smoothing across all frames was applied using a median Gaussian blur filter with a 1 to 2-pixel radius to reduce high background noise. Signal background offset corrections and scale bar additions attained from the camera calibration were then applied. Following image preprocessing, dual-channel image stacks were merged to facilitate colocalization analysis. Colocalized signals in the merged channels for particle analysis, the *"AND"* operation on Image J was performed on using the *"Imaging Calculator"* function. This generated a new image stack displaying only the overlapping signals between the two channels. The resulting image stack was then converted to 8-bit grayscale for automatic thresholding and subsequent particle analysis. Automatic thresholding was applied and defined for distinguishing the signal from the background for which the Moments method *(Image > Adjust > Threshold… > Moments)* was selected. The background mode was set to dark and converted to a binary mask to enable colocalized signals from the merged image stack to appear white on the black background, suitable for particle analysis and data export. Within the binary stack particle analysis parameters were set to distinguish particles from the background ($> 3\ \mu m^2$) to avoid counting artefacts or noise and a data summary created for further analysis.

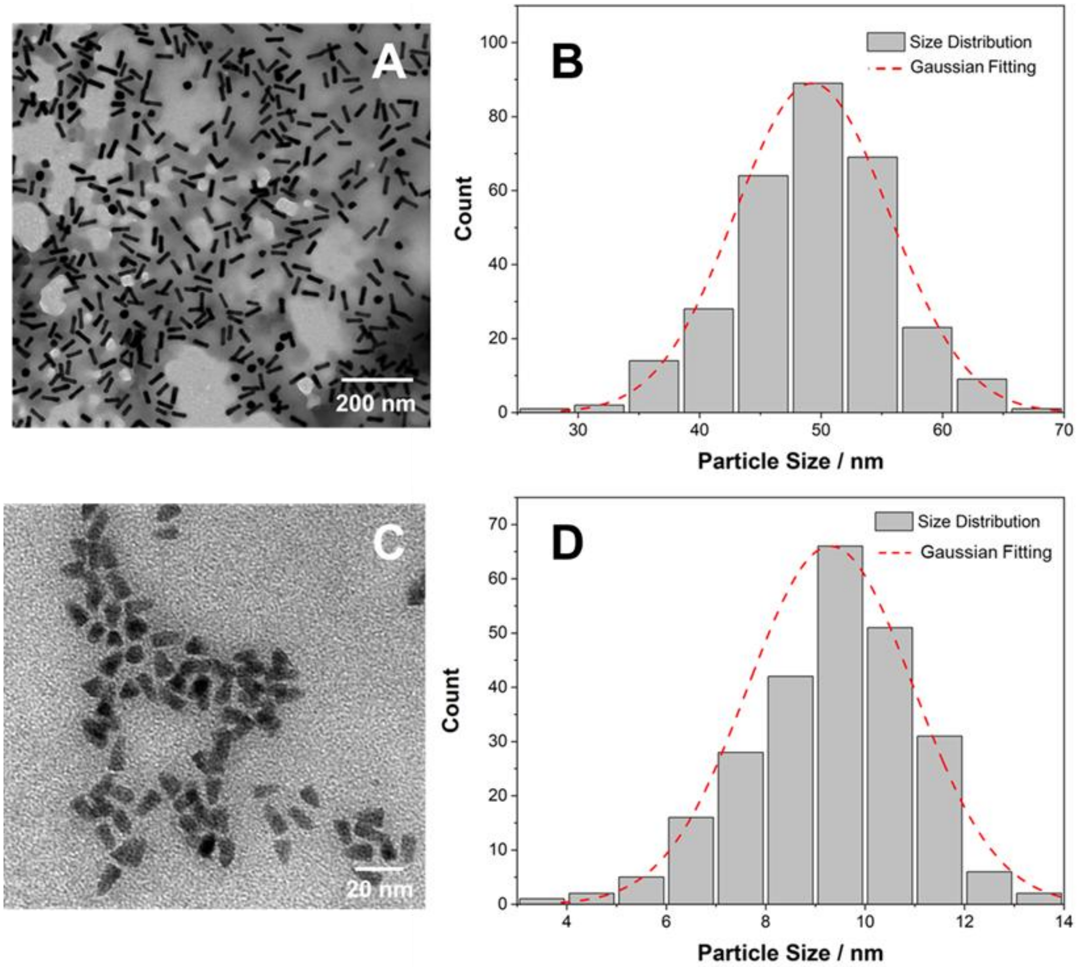

Figure S4. Transmission Electron Microscopy (TEM) Images of a) bare AuNRs and c) carboxylated QD655 particles with their repesective size distribution histrograms focusing on the longest axis plotted in b) AuNRs and d) QD's.

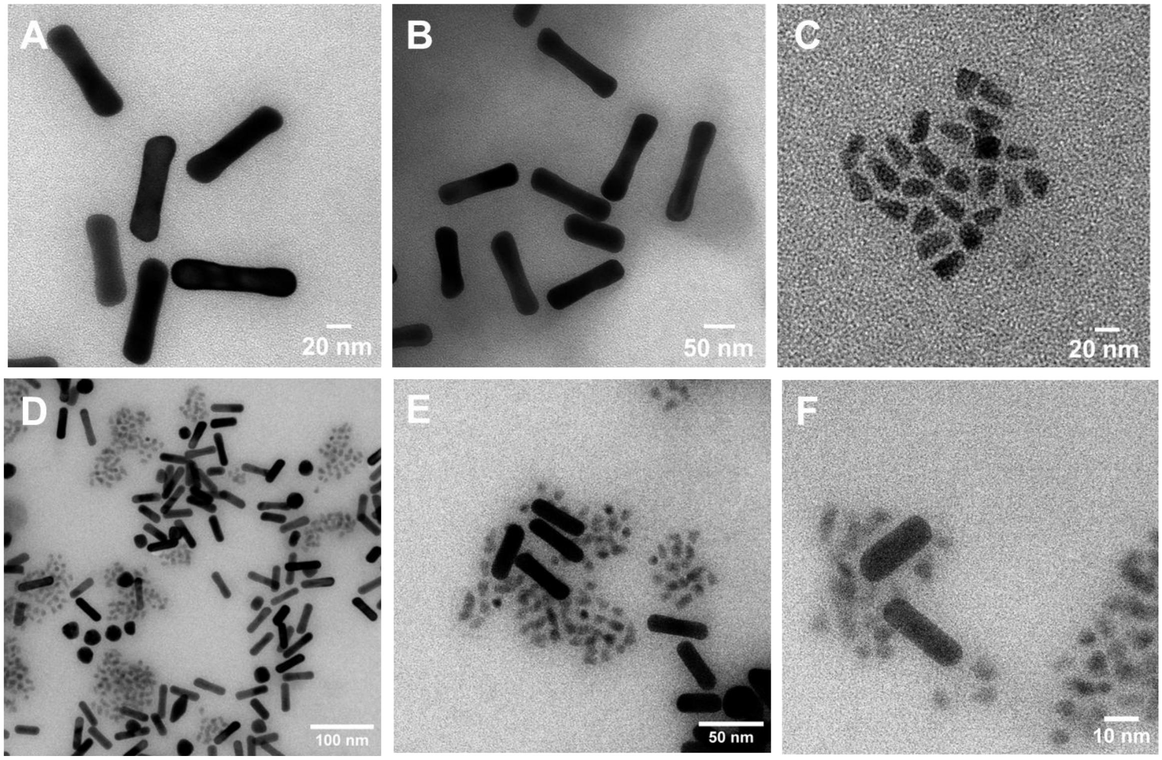

Figure S5. Transmission Electron Microscopy (TEM) Images of (A) CTAB NR, (B) PTA NR, (C) Indivdiual QD655 and (D – F) NR – QD assemmblies at various magifications. Scale bar: 10 – 100 nm.

A

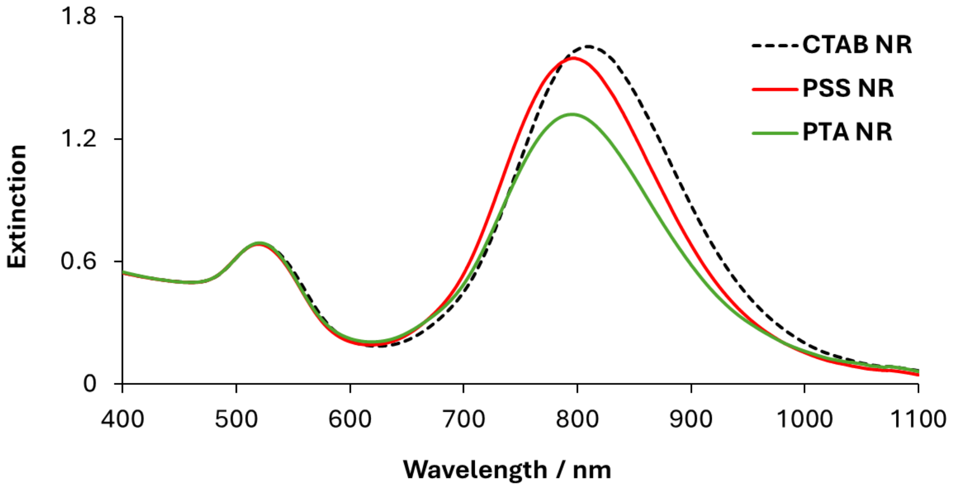

B

Surface Modified AuNR	Normalized λ_{max} / nm	Normalized O.D	Average Zeta Potential / mV
CTAB NR	809	1.65	+ 32.1
PSS NR	798	1.60	- 26.9
PTA NR	796	1.32	- 21.9

Figure S6. Characterization of functionalized NRs demonstrating pTA growth on negatively charged surfaces (i.e. PSS coated NRs) using (A) UV-Vis spectroscopy (normalized at 450 nm), and (B) corresponding UV-Vis data and zeta potentials showing particle stability post-functionalization. The original CTAB NRs were coated with PSS followed by pTA using the same conditions described in the Experimental section in the main article.

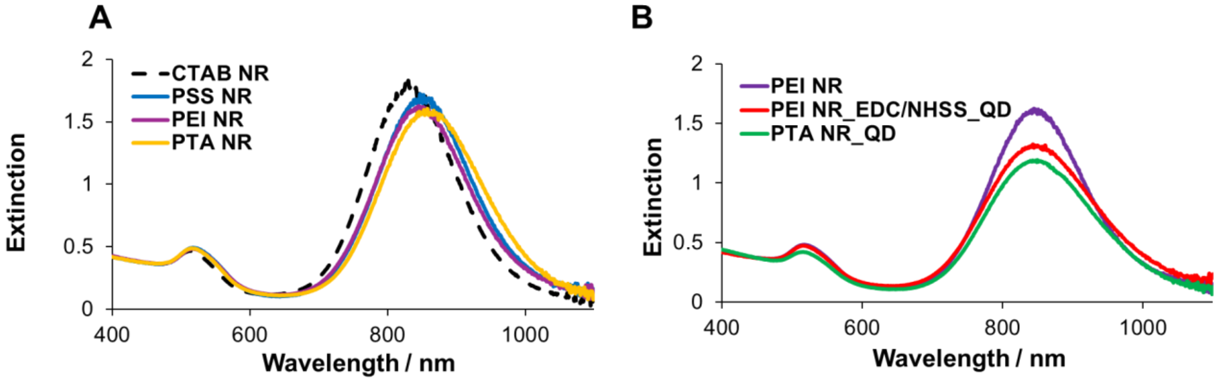

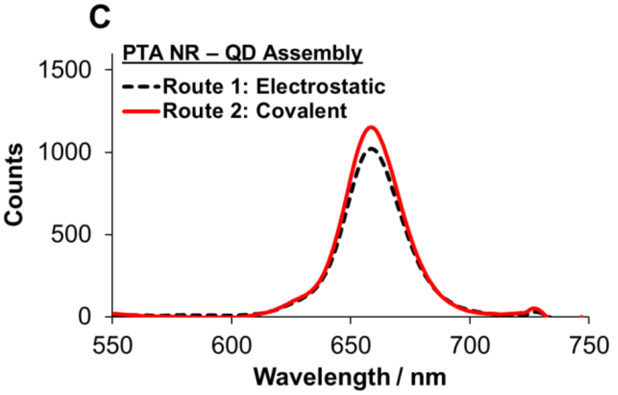

D

AuNR Surface Modification	Average Zeta Potential / mV
CTAB NR	+19.0
PSS NR	-36.0
PEI NR	+20.6
PTA NR	-21.6
PTA NR_QD	-23.1

Figure S7. Characterisation of covalent Route 2, showing similar results to the electrostatic Route 1: (A) UV-Vis of NR functionalisation, (B) UV-Vis of NR–QD assembly using EDC-NHSS coupling , (C) Bulk fluorescence spectra of the final NR–QD assembly indicating comparable signal intensities (at similar assemly conncetrations) between Routes 1 and 2. (D) Summary of average zeta potentials for each functionalized layer.

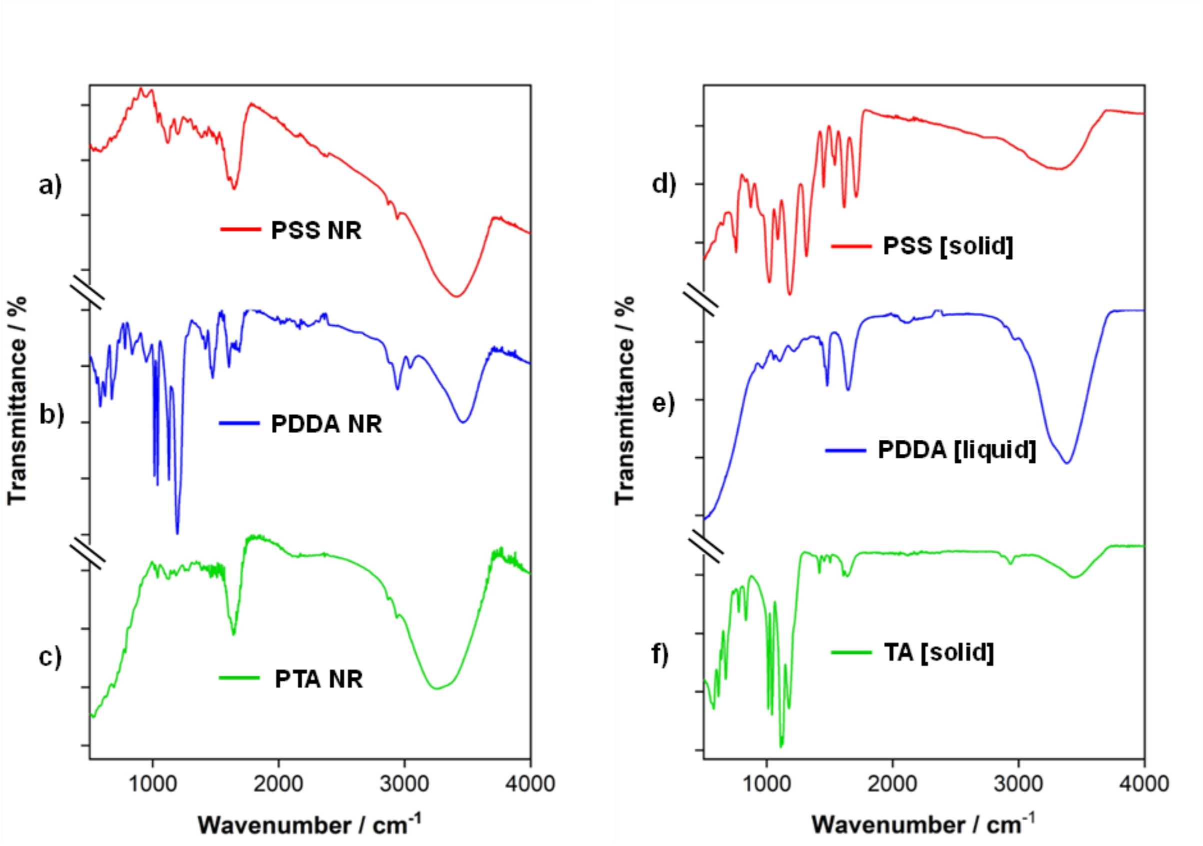

Figure S8. FTIR measurements supporting Route 1 (electrostatic): (a) – (c) functionalisation of NRs after each passivation step, and (d) – (f) controls of each polymer for confirmation of successful coatings on NRs.

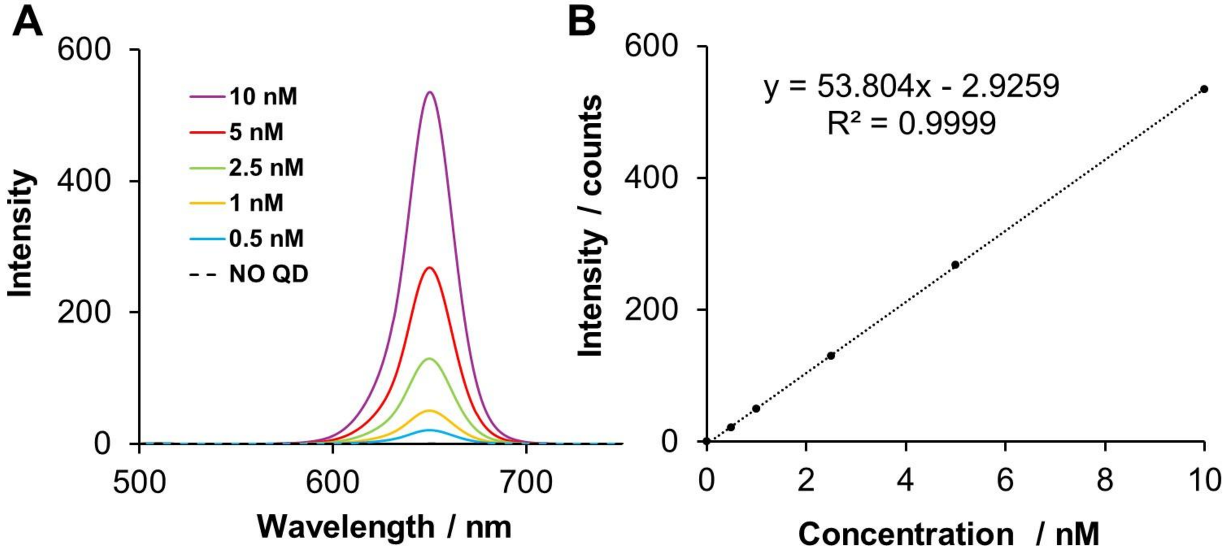

Figure S9. (A) Fluorescence spectra of carboxyl-QD655's without AuNRs (0.5 – 10 nM) at 435 nm excitation, serving as controls to demonstrate the linear relationship between fluorescence intensity and QD concentrations loaded onto surface modifed nanorods. (B) Calibration curve for fluorescence intensities of QD655 dilutions (0.5 – 10 nM).

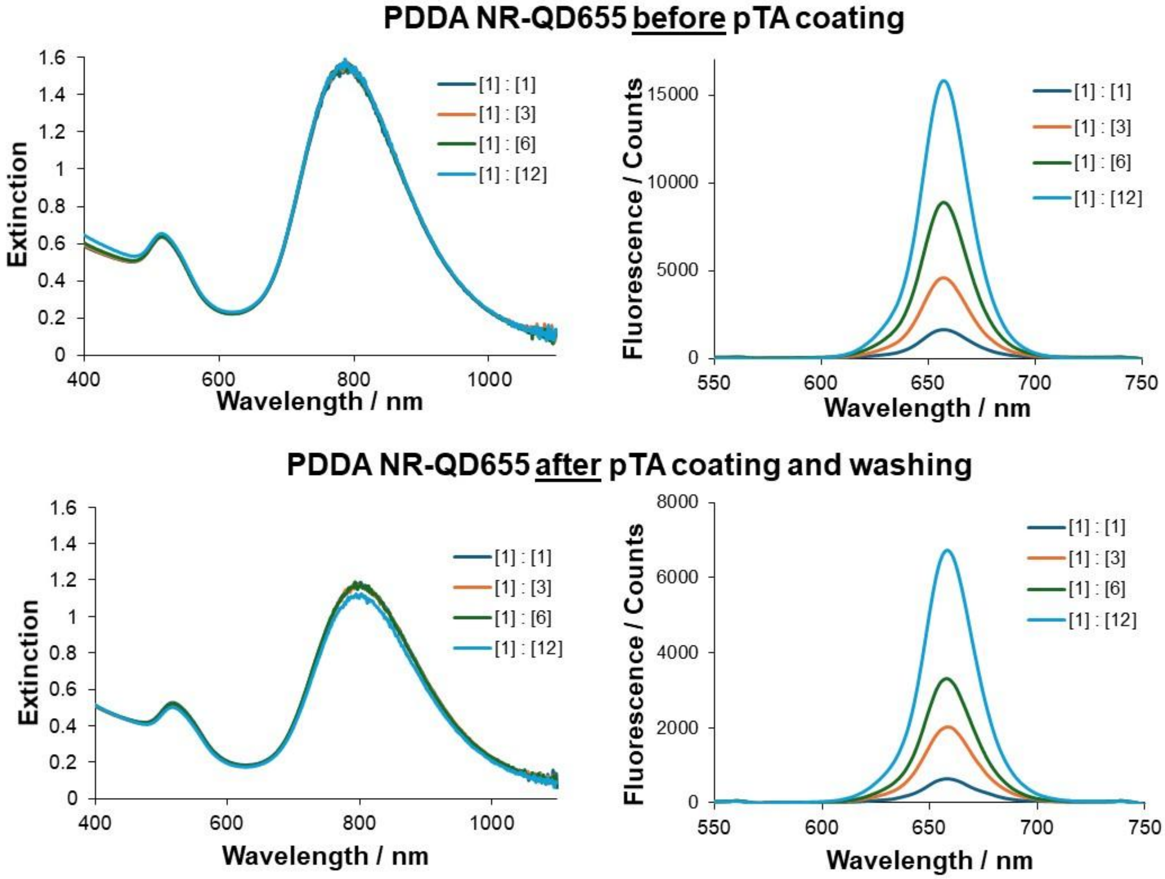

Figure S10. Comparison of NR-QD assemblies before and after the pTA coating step. Both extinction and bulk fluorescence spectra were acquired for a series of [NR]:[QD] concentration ratios. In both cases, the data was obtained after repeat washing to remove excess reagents and enable a comparison. The results show a typical red-shift of the NR LSPR λ_{max} of ~12-13 nm and damping of ~20% upon pTA coating while the QD655 emission λ_{max} is red-shifted by ~2 nm and the intensity is lowered by ~50%.

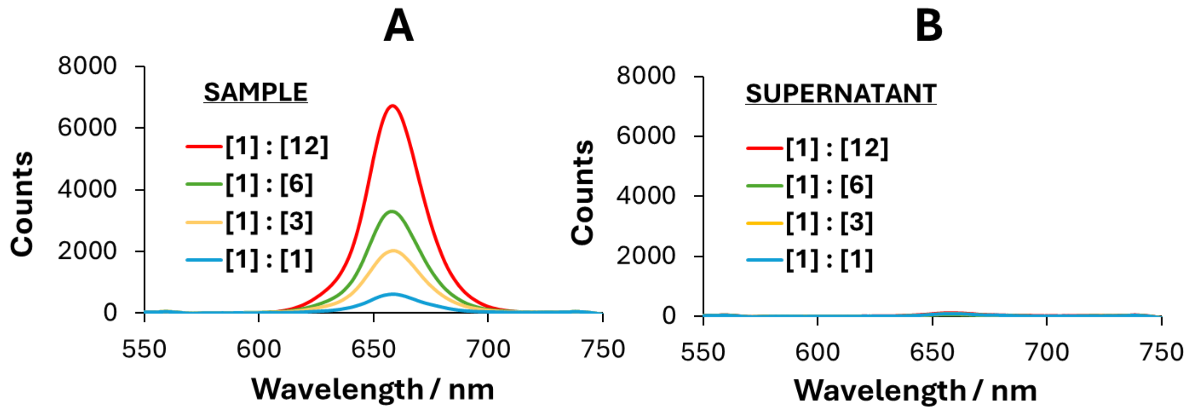

Figure S11. Fluorescence spectroscopy of (A) NR–QD colloidal samples and (B) their respective supernatants after the final centrifugal wash prepared via electrostatic Route 1. Each [NR]:[QD] loading ratio was analysed to confirm the removal of excess QDs. All spectra were acquired under identical conditions with 435 nm excitation.

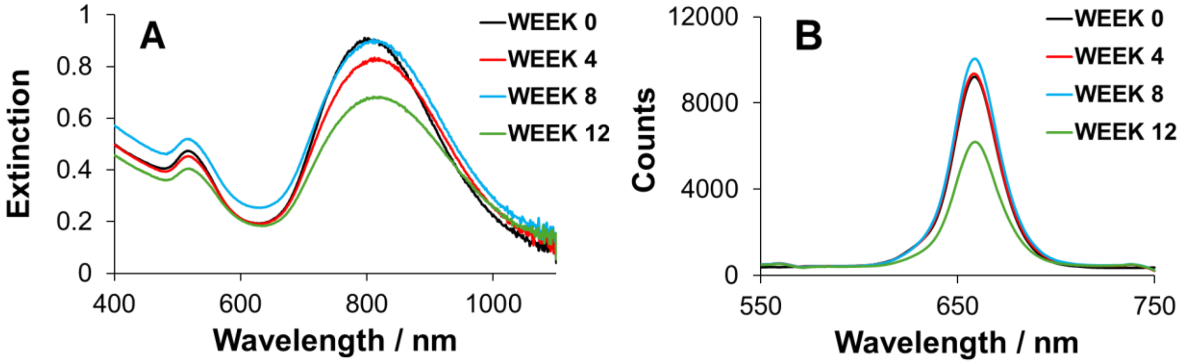

Figure S12. Three-month stability study of NR– QD stability assemblies at the highest loading ratio, [1]:[12], using (A) UV–Vis spectroscopy and (B) fluorescence imaging spectroscopy. Data was collected on the electrostatic Route 1 with a fixed nanorod concentration of 0.3 nM under identical acquisition conditions.

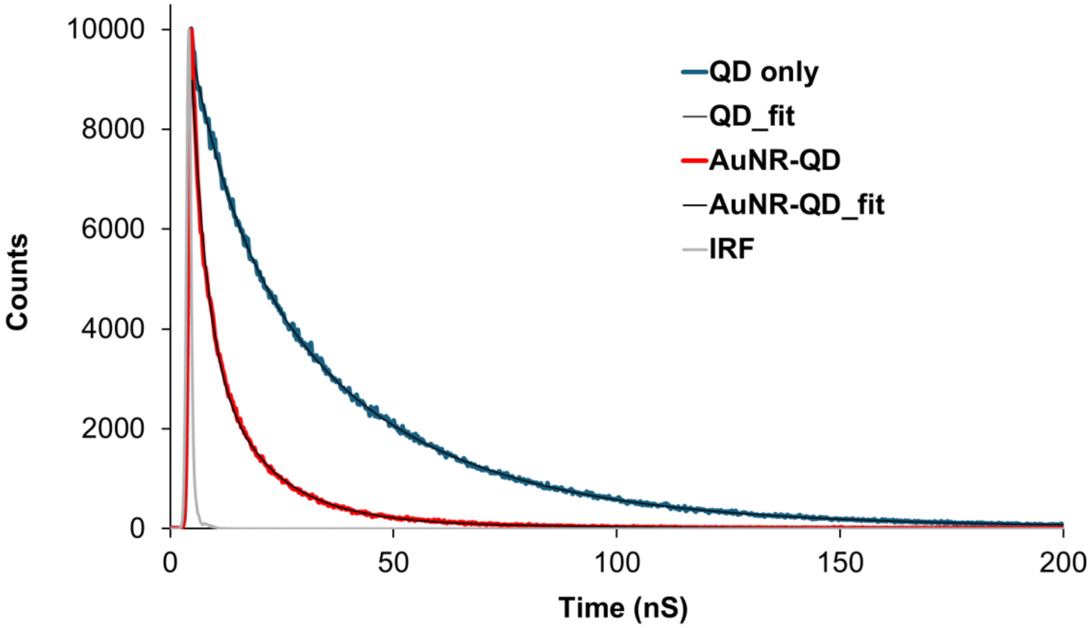

Figure S13. Photoluminescence lifetime measurements of both QD655 only (5 nM) and optimized PTA wrapped NR-QD assemblies (0.2 nM). These measurements were aquired with an Edinburgh Instruments FLS 1000 photoluminescence fluorimeter, λ_{ex} = 430 nm, λ_{em} = 655 nm in a 1 cm pathlength cuvette. A 5 nm bandwith for the emission collection was applied in both cases with the excitation bandwith adjusted from 8 nm for the QD only to 12 nm for the AuNR-QD's to obtain a similar signal count level. The instrument response function (IRF) was measured with Ludox.

Both PL emission curves have been fitted to a three exponential decay function:

$$Fit = A + B_1\, e^{(-t/\tau_1)} + B_2\, e^{(-t/\tau_2)} + B_3\, e^{(-t/\tau_3)}$$

	τ_1 (nS)	τ_2 (nS)	τ_3 (nS)	**$<\tau>_{amp}$ (nS)**	**$<\tau>_{int}$ (nS)**	χ^2
QD	9.71	34.37	75.46	**30.14**	**38.82**	1.074
AuNR-QD	2.98	12.88	46.08	**6.94**	**14.10**	1.292

The calculated Amplitude (B_i) Average Lifteime $<\tau>_{amp}$ and the Internsity Average Lifetime $<\tau>_{int}$ are also included here for comparison. The χ^2 values close to 1 indicate the good quality of the decay fit.

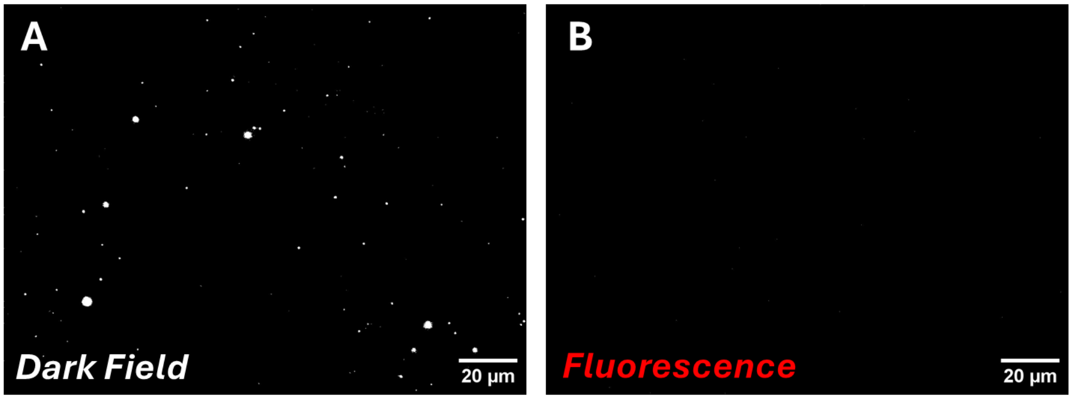

Figure S14. Dual imaging of NRs in the absence of QDs on a glass coverslip with the same area imaged by (A) dark field / Rayleigh scattering and (B) fluorescence. Scale bar = 20 µm for both images.

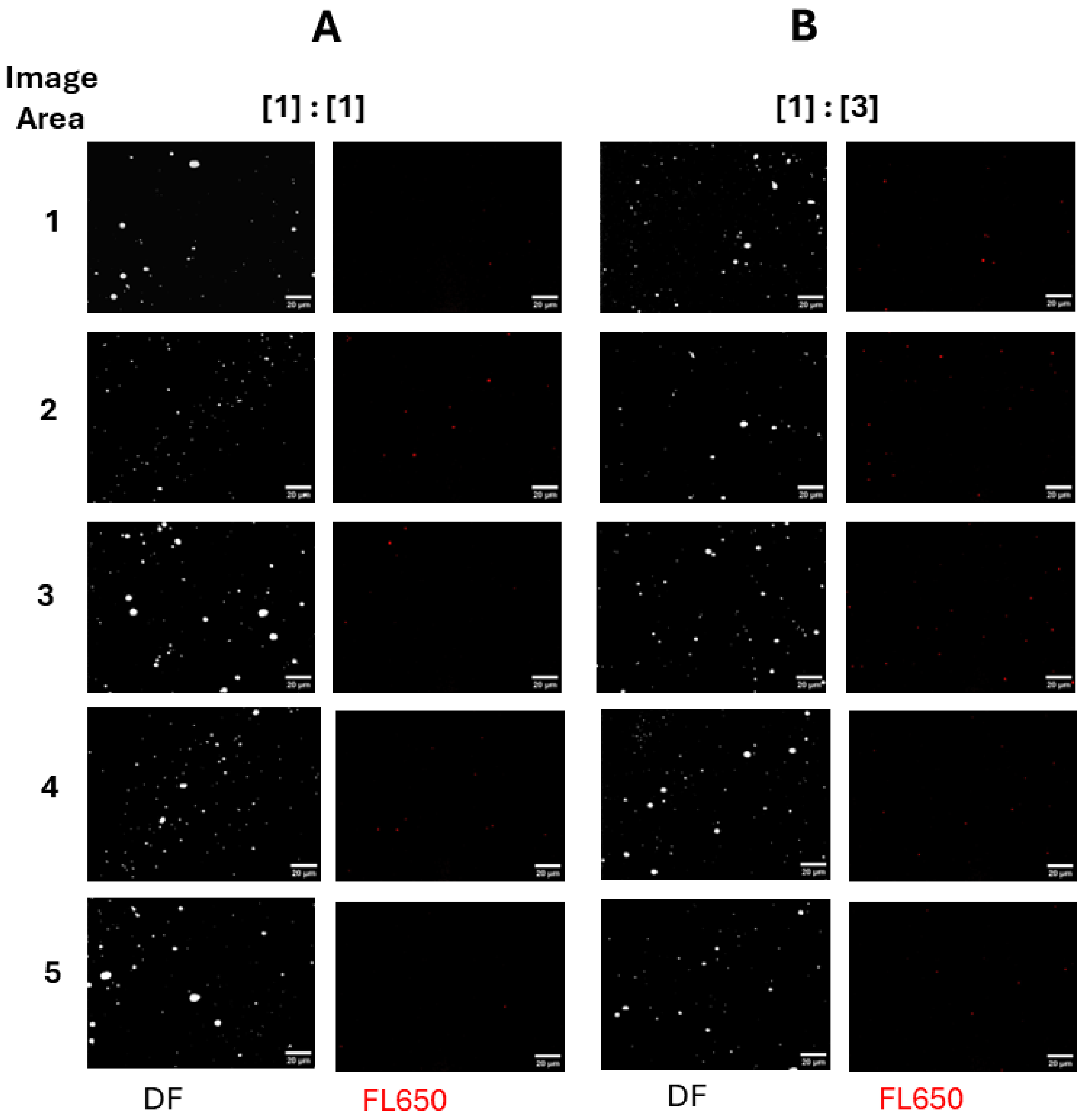

Figure S15 A&B. Dual imaging of NR-QD assemblies immobilised on glass coverslips across 5 different substrates, with the same area imaged by Dark field (DF)/ Rayleigh scattering and QD Fluorescence (FL650). Samples were prepared at a [AuNR]:[QD] concentration ratio A) [1]:[1] and B) [1]:[3]. Scale bar =20 μm for all images.

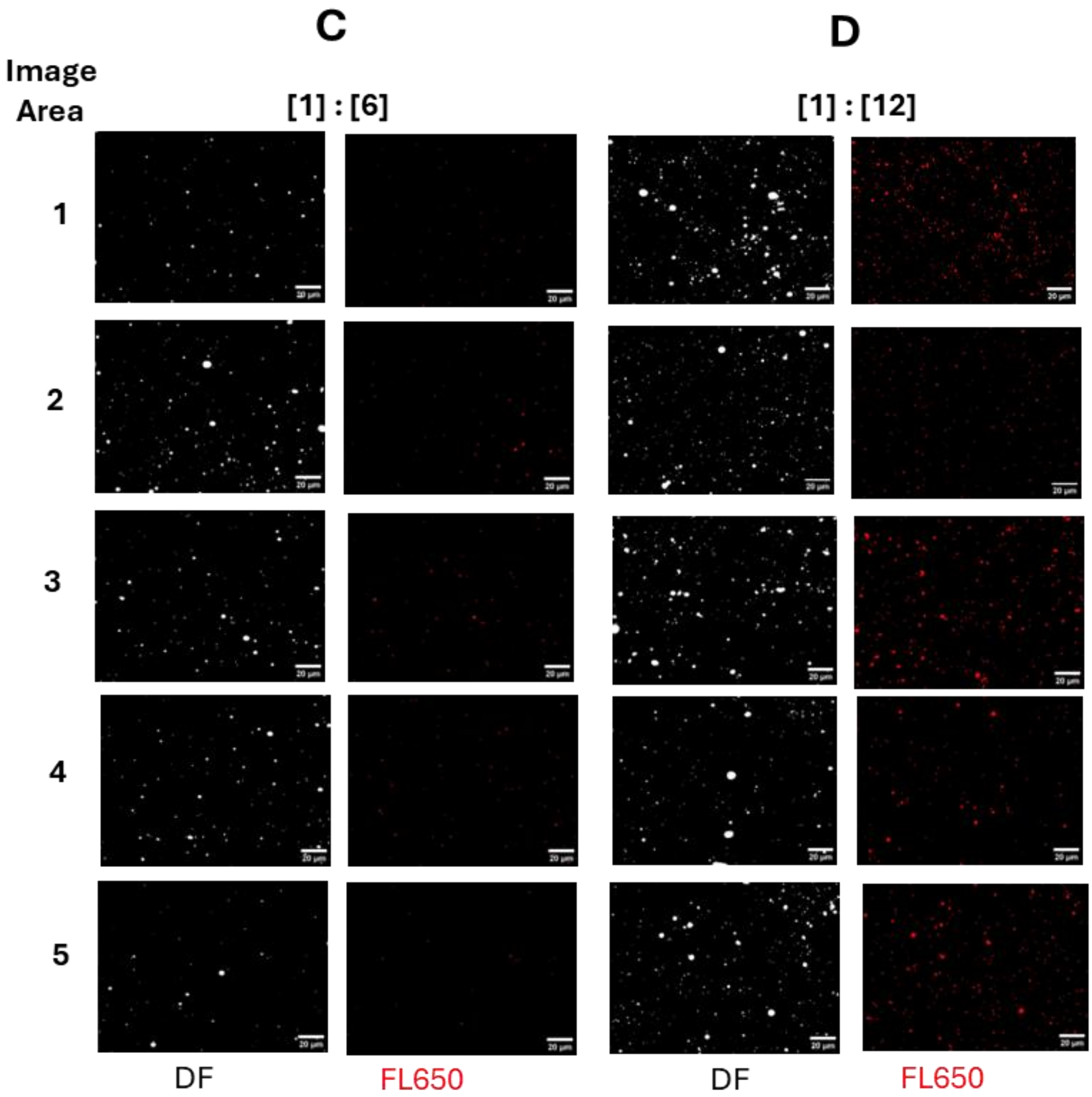

Figure S15 C&D. Dual imaging of NR-QD assemblies immobilised on glass coverslips across 5 different substrates, with the same area imaged by Dark field (DF)/ Rayleigh scattering and QD Fluorescence (FL650). Samples were prepared at a [AuNR]:[QD] concentration ratio C) [1]:[6] and D) [1]:[12]. Scale bar =20 µm for all images.

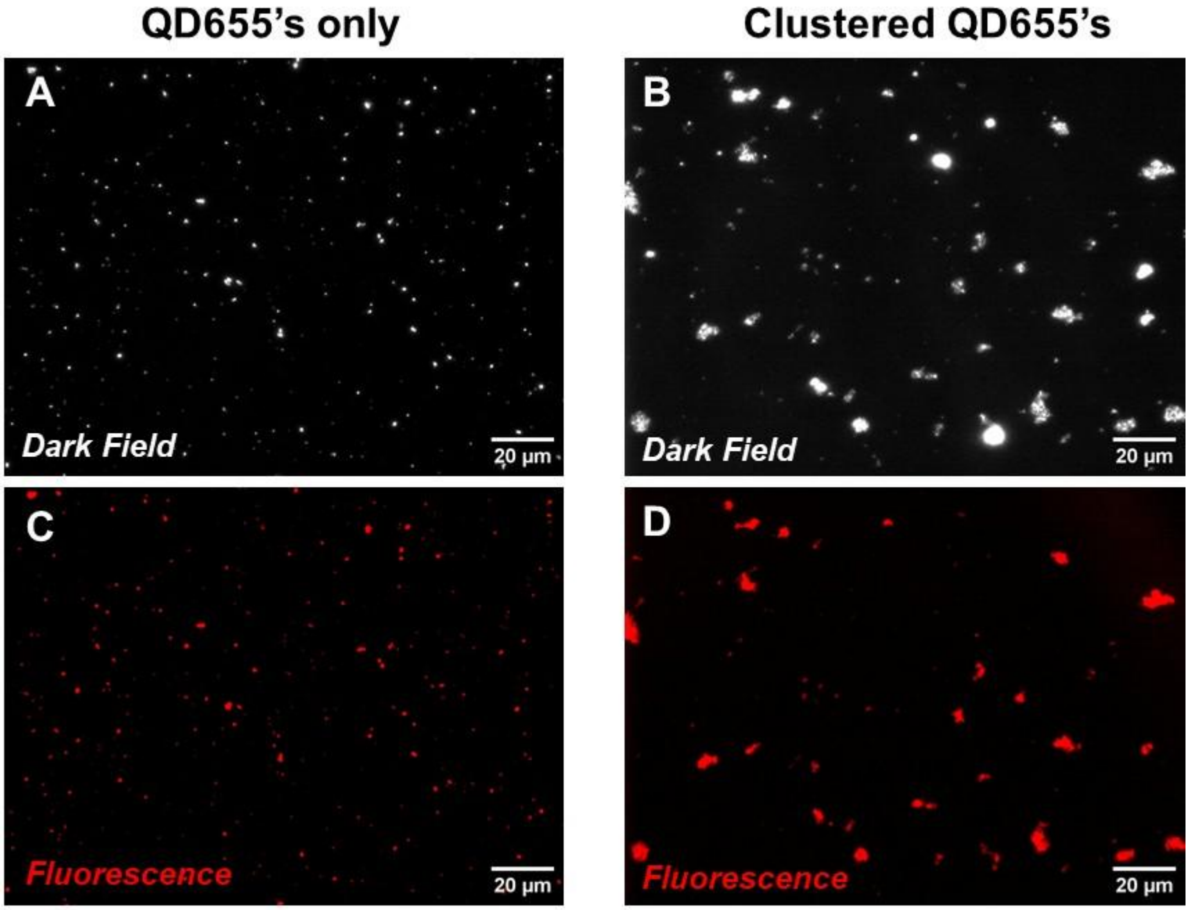

Figure S16. Dual imaging of QD655’s and QD655 clusters immobilized on PEI coated glass coverslips. QD655’s only (with no induced aggregation) were deposited at a low stock concentration of 0.008 nM with the slide then gently rinsed and N_2 dried to minimise drying-induced aggregation. The QD clusters were formed through tannic acid polymerisation and then high-speed centrifugation before covering a fresh coverslip. In both cases, the same area is imaged using (A) dark field / Rayleigh scattering and (C) fluorescence for QD655’s only, and (B) dark field / Rayleigh scattering and (D) fluorescence for the QD clusters. Fluorescence was captured at 435/40 nm excitation, 514 nm dichroic, and 650/60 nm emission. Scale bar = 20 µm for all images.

Supporting Tables

Table S1. UV–Vis and NTA analysis obtained from Figure 2 for the characterisation of surface modified AuNRs for Route 1 electrostatic based method. The average zeta potentials for each custom surface modification are also reported.

AuNR Surface Modifications	λ_{max} / nm	Normalized O.D	Average Zeta Potential / mV	Hydrodynamic Diameter / nm
CTAB NR	830	1.8	+32.1	45
PSS NR	838	1.9	–26.9	44
PDDA NR	856	1.8	+21.9	69
PTA NR (Route 1)	858	1.6	–29.9	86

Table S2. Dynamic Light Scattering and zeta-potential measurements performed on individual QDs and clustered QDs (carboxyl functionalised QD655) induced by tannic polymerisation, highlighting the changes in particle characteristics and behavior during the formation of NR–QD assemblies.

Carboxyl QD655	Peak 1 Average (d.nm)	Peak 2 Average (d.nm)	Peak 3 Average (d.nm)	Peak 1 Area (Percent)	Peak 2 Area (Percent)	Peak 3 Area (Percent)	Size / nm	Zeta Potential / mV
**Individual**	256.9	1707.8	820.7	96.1	3.0	0.9	25.7	-32.0
**Clustered**	446.8	3159.8	22.7	73.2	17.9	8.9	44.7	-15.1

Table S3. Details of UV-VIS and fluorescence analysis for NR–QD assemblies in colloidal solution in Figure 3. QD loading ratios were increased from [1]:[1] to [1]:[12], with the NR concentration fixed to 0.3 nM. The table includes analysis of the UV-VIS spectra: λ_{max}, optical densities (ODs) as well as the bulk fluorescence emission λ_{max} and intensities at 435 nm excitation for each sample.

**[AuNR] : [QD]**	**λ_{max} / nm**	**Normalized O.D**	**Emission λ_{max} / nm**	**Emission Intensity**
No QD	858	1.3	N/A	N/A
[1] : [1]	856	0.87	653	17.4
[1] : [3]	855	0.91	653	72.4
[1] : [6]	857	0.84	654	169.8
[1] : [12]	860	0.96	654	299.1

Table S4. Zeta potentials and hydrodynamic diameters reported for NR–QD assemblies with QD loading ratios ranging from [1]:[1] to [1]:[12], while NR concentration was fixed at 0.3 nM.

**[AuNR] : [QD]**	**Zeta Potential / mV**	**Average Zeta Potential / mV**	**Average Hydrodynamic Diameter / nm**
No QD	-29.9	-29.9	86
[1] : [1]	-24.3	-23.8	77
[1] : [3]	-27		
[1] : [6]	-22.5		
[1] : [12]	-21.4		

Table S5. NR–QD conjugation efficiency (%) from dual imaging (Figure S12) of single nanoparticles immobilised on glass coverslips for NR – QD assemblies at different QD loading ratios. Five different substrate areas were assessed and co-localised signals counted.

[AuNR] : [QD]	Image Area	Conjugation Performance / %	Average Conjugation Efficiency / %
[1] : [1]	1	7	13
	2	11	
	3	15	
	4	17	
	5	18	
[1] : [3]	1	28	48
	2	70	
	3	57	
	4	47	
	5	38	
[1] : [6]	1	90	87
	2	90	
	3	76	
	4	92	
	5	86	
[1] : [12]	1	93	93
	2	93	
	3	92	
	4	90	
	5	96	

Table S6. Assessing conjugation efficiency from dynamic dual imaging analysis of NR–QD assemblies at the highest loading density ([1]:[12]).

Video	ANDOR 885<br>No. of Rayleigh Scattered Particles Identified	ANDOR 897<br>No. of Fluorescence Particles Identified	Conjugation Efficiency/ %
1	20	17	85
2	9	9	100
3	17	16	94
4	16	16	100
5	13	13	100
6	10	9	90
7	20	20	100
8	24	24	100
9	22	22	100
10	21	21	100
		**AVERAGE**	**96.9%**

Table S7. Assessing conjugation efficiency from dynamic dual imaging analysis of NR–QD assemblies at the lowest loading density ([1]:[1]).

Video	ANDOR 885<br>No. of Rayleigh Scattered Particles Identified	ANDOR 897<br>No. of Fluorescence Particles Identified	Conjugation Efficiency/ %
1	17	8	47
2	14	7	50
3	16	9	56
4	16	7	44
5	14	8	57
6	18	9	50
7	14	8	57
8	9	5	56
		**AVERAGE**	**52 %**

Supporting Videos

Real time dual-modal videos representing both Rayleigh scattering and fluorescence channels are provided in gif. format and can be accessed electronically. The file names are listed below with each video including a scale bar of 20 μm. These videos correspond to the dynamic imaging results discussed in the main text and illustrate individual nanoparticles moving freely in suspension. In each case the Rayleigh scattering imaging is shown on the left (white particles on a dark background) and the fluorescence imaging on the right (red particles on a dark background).

Video S1: *NRQD655_High Loading_Electrostatic Method_Dual Channels*

Video S2: *NRQD655_ High Loading_Covalent Method_Dual Channels*

Video S3: *Individual QD655_Dual Channels*

Video S4: *Cluster QD655_Dual Channels*
